# Circular RNA *circCORO1C* promotes laryngeal squamous cell carcinoma progression by modulating the let-7c-5p/PBX3 axis

**DOI:** 10.1186/s12943-020-01215-4

**Published:** 2020-06-02

**Authors:** Yongyan Wu, Yuliang Zhang, Xiwang Zheng, Fengsheng Dai, Yan Lu, Li Dai, Min Niu, Huina Guo, Wenqi Li, Xuting Xue, Yunfeng Bo, Yujia Guo, Jiangbo Qin, Yixiao Qin, Hongliang Liu, Yu Zhang, Tao Yang, Li Li, Linshi Zhang, Rui Hou, Shuxin Wen, Changming An, Huizheng Li, Wei Xu, Wei Gao

**Affiliations:** 1 Shanxi Key Laboratory of Otorhinolaryngology Head and Neck Cancer, Shanxi Medical University, Taiyuan, 030001 Shanxi People’s Republic of China; 2 Shanxi Province Clinical Medical Research Center for Precision Medicine of Head and Neck Cancer, The First Hospital of Shanxi Medical University, Taiyuan, 030001 Shanxi People’s Republic of China; 3 Department of Otolaryngology Head & Neck Surgery, The First Hospital of Shanxi Medical University, Taiyuan, 030001 Shanxi People’s Republic of China; 4 Key Laboratory of Cellular Physiology, Ministry of Education, Shanxi Medical University, Taiyuan, 030001 Shanxi People’s Republic of China; 5 Department of Biochemistry & Molecular Biology, Shanxi Medical University, Taiyuan, 030001 Shanxi People’s Republic of China; 6 Department of Otolaryngology Head & Neck Surgery, The First Hospital, Jinzhou Medical University, Jinzhou, 121001 Liaoning People’s Republic of China; 7 Department of Pathology, Shanxi Cancer Hospital, Shanxi Medical University, Taiyuan, 030013 Shanxi People’s Republic of China; 8 Department of Otolaryngology Head & Neck Surgery, Heping Hospital Affiliated to Changzhi Medical College, Changzhi, 046000 Shanxi People’s Republic of China; 9 Department of Cell Biology and Genetics, Basic Medical School of Shanxi Medical University, Taiyuan, 030001 Shanxi People’s Republic of China; 10 Department of Physiology, Shanxi Medical University, Taiyuan, 030001 Shanxi People’s Republic of China; 11 Department of Hepatobiliary and Pancreatic Surgery, The Second Affiliated Hospital, Zhejiang University School of Medicine, Hangzhou 310009 Zhejiang, People’s Republic of China; 12 Harry Perkins Institute of Medical Research, QEII Medical Centre and Centre for Medical Research, the University of Western Australia, PO Box 7214, 6 Verdun Street, Nedlands, Perth, Western Australia 6009 Australia; 13 General Hospital, Shenzhen University, Shenzhen, 518055 Guangdong People’s Republic of China; 14 Department of Head and Neck Surgery, Cancer Hospital, National Cancer Center, Chinese Academy of Medical Sciences & Peking Union Medical College, Beijing, 100021 People’s Republic of China; 15 Department of Otolaryngology Head & Neck Surgery, Dalian Municipal Friendship Hospital, Dalian Medical University, Dalian, 116100 Liaoning People’s Republic of China; 16 Shandong Provincial ENT Hospital Affiliated to Shandong University, Jinan, 250022 Shandong People’s Republic of China; 17 Shandong Provincial Institute of Otolaryngology, Jinan, 250022 Shandong People’s Republic of China; 18 Key Laboratory of Otolaryngology, Ministry of Health, Shandong University, Jinan, 250022 Shandong People’s Republic of China

**Keywords:** circCORO1C, Let-7c-5p, PBX3, Laryngeal squamous cell carcinoma, Epithelial–mesenchymal transition, Metastasis

## Abstract

**Background:**

Laryngeal squamous cell carcinoma (LSCC) is a common malignant tumor of the head and neck. LSCC patients have seriously impaired vocal, respiratory, and swallowing functions with poor prognosis. Circular RNA (circRNA) has attracted great attention in cancer research. However, the expression patterns and roles of circRNAs in LSCC remain largely unknown.

**Methods:**

RNA sequencing was performed on 57 pairs of LSCC and matched adjacent normal mucosa tissues to construct circRNA, miRNA, and mRNA expression profiles. RT-PCR, qPCR, Sanger sequencing, and FISH were undertaken to study the expression, localization, and clinical significance of *circCORO1C* in LSCC tissues and cells. The functions of *circCORO1C* in LSCC were investigated by RNAi-mediated knockdown, proliferation analysis, EdU staining, colony formation assay, Transwell assay, and apoptosis analysis. The regulatory mechanisms among *circCORO1C*, *let-7c-5p*, and *PBX3* were investigated by luciferase assay, RNA immunoprecipitation, western blotting, and immunohistochemistry.

**Results:**

*circCORO1C* was highly expressed in LSCC tissues and cells, and this high expression was closely associated with the malignant progression and poor prognosis of LSCC. Knockdown of *circCORO1C* inhibited the proliferation, migration, invasion, and in vivo tumorigenesis of LSCC cells. Mechanistic studies revealed that *circCORO1C* competitively bound to *let-7c-5p* and prevented it from decreasing the level of *PBX3*, which promoted the epithelial–mesenchymal transition and finally facilitated the malignant progression of LSCC.

**Conclusions:**

*circCORO1C* has an oncogenic role in LSCC progression and may serve as a novel target for LSCC therapy. *circCORO1C* expression has the potential to serve as a novel diagnostic and prognostic biomarker for LSCC detection.

## Background

Laryngeal squamous cell carcinoma (LSCC) is a common malignant tumor of the head and neck originating from the laryngeal mucosal epithelium. LSCC accounts for approximately 2.4% of systemic malignancies worldwide each year; in 2018, around 95,000 people died of laryngeal cancer [1]. The onset of LSCC is occult, and approximately 60% of patients are in the advanced stages when diagnosed (clinical stages III and IV) [2]. The proneness of LSCC to local invasion and cervical lymph node metastasis seriously interferes with patient survival rates [3]. Surgery remains the main treatment approach for LSCC [4]. Unfortunately, LSCC is one of the few tumors with a decreasing survival rate in recent years, and its 5-year survival rate has declined from 66 to 63% over the past 40 years [5], which is mainly attributed to its unclear mechanism of occurrence and progression. Therefore, it is urgent to reveal the pathogenesis of LSCC, identify biomarkers for its diagnosis, and investigate effective new therapeutic targets.

Circular RNA (circRNAs) is a recently identified non-coding RNA that has become the latest hotspot in cancer research. The circRNA molecule has a closed loop structure that is not affected by exonucleases and is not easily degraded. circRNAs also have features of high conservation and abundance [6]. Hence, circRNAs have unique advantages as biomarkers for disease diagnosis and prognosis. Recent studies have shown that circRNA molecules are rich in miRNA binding sites and can specifically bind miRNAs, thereby eliminating the inhibitory effect of miRNAs on target genes and upregulating the expression level of target genes, that is, functioning as competing endogenous RNA (ceRNA) [7]. circRNAs also bind to RNA binding proteins and may translate proteins to exert their functions [8, 9]. circRNAs have critical regulatory effects in the occurrence and development of a variety of cancers, affecting cell cycle, apoptosis, metabolism, invasion, and metastasis [10]. *circAGFG1* upregulates *CCNE1* expression and promotes the proliferation, migration, and invasion of breast cancer cells [11]. *circPPP1R12A*-encoded protein circPPP1R12A-73aa promotes tumor growth and metastasis of colon cancer [12]. However, to date, little is known about the expression, functions, and regulatory mechanisms of circRNAs in LSCC.

Pre-B-cell leukemia homeobox transcription factor 3 (PBX3) is a member of the evolutionarily conserved three-amino acid-loop-extension (TALE) homeodomain transcription factor family. A recent study revealed that PBX3 is a critical regulatory protein of the epithelial–mesenchymal transition (EMT) network in colorectal cancer [13]. Dysregulation of PBX3 expression has been observed in many cancer types, such as prostate, gastric, cervical, and liver cancer [14–17]. Nonetheless, the expression and function of PBX3 in LSCC are still unknown.

In this study, we performed RNA sequencing of 57 pairs LSCC tissues and matched adjacent normal mucosa (ANM) tissues and identified abnormally upregulated expression of *circCORO1C* in LSCC tissues. Furthermore, the expression of *circCORO1C* was strongly associated with the clinical features and prognosis of LSCC patients. We found that *circCORO1C* could bind to *let-7c-5p* and prevent it from decreasing the level of PBX3, which promoted EMT and stimulated the proliferation, migration, and invasion of LSCC cells in vitro and in vivo.

## Methods

### LSCC patient tissue

A total of 164 pairs of LSCC tissues and matched ANM tissues (taken 1–3 cm from the edge of cancer tissues) were obtained from patients undergoing surgery at the Department of Otolaryngology Head and Neck Surgery, The First Hospital of Shanxi Medical University, from January 2013 to January 2017. None of the patients received chemotherapy or radiotherapy before surgery. The tissue samples were diagnosed independently by two experienced clinical pathologists. The histological types of LSCC were determined according the World Health Organization (WHO) system, and TNM (Tumor, Node, Metastasis) stage was defined according to the criteria of the American Joint Committee on Cancer (AJCC, 8th edition). Fresh specimens were immediately frozen in liquid nitrogen. Among the 164 pairs of tissue samples, 57 paired LSCC (Additional file 1: Table S1) and ANM tissues were used for RNA sequencing, and 107 paired samples for qPCR analysis (Additional file 1: Table S2).

### Cell lines and cell culture

Human LSCC cell line FD-LSC-1 (a gift from Professor Liang Zhou [18]) was cultured in BEGM™ Bronchial Epithelial Cell Growth Medium (Lonza, Walkersville, MD, USA) supplemented with 10% FBS (Biological Industries, CT, USA). Human LSCC cell line TU-177 purchased from Bioleaf Biotech Corporation (Shanghai, China) was maintained in DMEM supplemented with 10% FBS. Human HEK293T and MRC-5 cell lines were purchased from the China Center for Type Culture Collection (CCTCC). HEK293T cells were cultured in DMEM with 10% FBS. MRC-5 cells were cultured in MEM with 10% FBS. Human oral keratinocytes (HOK) purchased from ScienCell Research Laboratories (Carlsbad, CA) were cultured in DMEM with 10% FBS. All cells were cultured at 37 °C with 5% CO_2_. Cell lines were tested for mycoplasma contamination using the TransDetect PCR Mycoplasma Detection Kit (TransGen Biotech, Beijing, China).

### RNA and genomic DNA (gDNA) extraction

Total RNA was extracted from tissues or cells using Trizol reagent (Invitrogen, Waltham, MA) following the manufacturer’s instructions. The nuclear and cytoplasmic fractions were extracted using a PARIS kit (ThermoFisher Scientific, Waltham, MA). gDNA was extracted using a genomic DNA isolation kit (TIANGEN Biotech (Beijing) Co., Ltd., Beijing, China).

### RNA sequencing analysis

The RNA integrity of 57 pairs of LSCC/matched ANM tissues was examined with a Bioanalyzer 2100 (Agilent, Santa Clara, CA). High-quality RNA (RIN > 7) samples were subjected to library construction, and then each library was sequenced on an Illumina HiSeq 4000 (circRNA and mRNA) and Illumina HiSeq 2000 (miRNA) following the standard procedures by Novogene (Beijing, China). RNA sequencing data were deposited at GEO and are accessible via accession numbers GSE127165 and GSE133632. Differentially expressed circRNAs, miRNAs, and mRNAs were screened as reported [19] (Additional file 1: Table S3–5).

### RT-PCR and quantitative real-time PCR (qPCR)

For PCR of mRNA and circRNA, RNA was reverse-transcribed using a HiScript II 1st Strand cDNA Synthesis Kit (Vazyme, Nanjing, China). For qPCR of miRNA, cDNA was synthesized using an All-in-One™ miRNA First-Strand cDNA Synthesis Kit (GeneCopoeia, Rockville, MD). qPCR was performed using ChamQ SYBR qPCR Master Mix (Vazyme, Nanjing, China) on an ABI Stepone Plus system. The relative expression levels were calculated using the 2 ^(−△△CT)^ method. The circRNA and mRNA levels were normalized by *18 s* rRNA. The miRNA level was normalized against *U6* small nuclear RNA. Primer sequences are listed in Additional file 1: Table S6.

### RNase R treatment

Total RNA (2 μg) was incubated for 10 min at 37 °C with or without 3 U/μg RNase R (Geneseed Biotech Co., Ltd., Guangzhou, China), followed by RNA purification using a RNeasy MinElute Cleanup kit (Qiagen, Hilden, Germany) and analyzed by RT-PCR.

### Agarose gel electrophoresis

PCR products were separated by 2% agarose gel electrophoresis with TAE buffer using a 100 bp DNA ladder (TransGen Biotech, Beijing, China). The bands were photographed under an Azure C600 imager (Azure Biosystems, Dublin, CA).

### Fluorescence in situ hybridization (FISH)

Cy3-labeled circCORO1C probes (5′- AGAGCAATTGGTTCCTGCATATTTTTCTGGCAATCTCACATTTGTTAACATC -3′) were synthesized by Sangon Biotech (Shanghai, China). FISH was performed using a FISH kit (RiboBio, Guangzhou, China) according to the manufacturer’s instructions. Nuclei were stained with DAPI. Images were acquired on a Leica TCS SP8 confocal laser scanning microscope (Leica Microsystems Inc., Buffalo Grove, IL).

### Plasmid construction and cell transfection

The PBX3 overexpression plasmid was generated by inserting *PBX3* CDS sequence into the p3 × FLAG-CMV-10 vector (Sigma-Aldrich, St. Louis, MO). shRNA lentiviral plasmid targeting *circCORO1C* (psh-*circCORO1C*) was constructed by inserting annealed shRNA template DNA sequence into the pLKO.1 vector. For luciferase reporter plasmids, the sequences of *circCORO1C*, wild type, and *let-7c-5p* binding site mutant *PBX3* 3′ UTR were cloned into the psiCHECK-2 vector (Promega, Madison, WI). Cells were transfected using Lipofectamine 3000 (Invitrogen) according to the manufacturer’s instructions.

### siRNAs, miRNA mimics, and inhibitor

siRNAs targeting *circCORO1C* (si-*circCORO1C* #1: 5′-AGAUUGCCAGAAAAAUAUGCA-3′; si-*circCORO1C* #2: 5′-UUGCCAGAAAAAUAUGCAGGA-3′), negative control siRNAs (si-NC), stable oligonucleotides (modified by cholesterol, 2′-OMe and phosphorothioate), *let-7c-5p* mimics, and NC were synthesized by GenePharma (Shanghai, China). miRNA inhibitor is small, chemically modified single-stranded RNA molecule that can competitively bind to and inhibit the function of specific endogenous mature miRNA. 2′-OMe-modified *let-7c-5p* inhibitor and NC inhibitor were synthesized by GenePharma.

### Generation of *circCORO1C* knockdown cells

To generate FD-LSC-1 cells with stable knockdown of *circCORO1C*, lentiviruses were produced in HEK293T cells by cotransfection with psh-*circCORO1C* and packaging plasmids GAG and VSVG. Virus supernatant was harvested 48 h after transfection, mixed with polybrene (8 μg/ml), and added to FD-LSC-1 cells. After 48 h incubation, 2 μg/ml puromycin (Santa Cruz Biotechnology, Dallas, TX) was added for 1 week to screen for stable cell clones.

### CCK8 assay

After 24 h transfection, cells were digested and seeded into 96-well plates (3 × 10^3^/well). At 0, 24, 48, 72, and 96 h after seeding, each well was replaced with 100 μL fresh complete medium and 10 μL TransDetect CCK (TransGen Biotech, Beijing, China) followed by incubation at 37 °C with 5% CO_2_ for 1 h. The absorbance of the solution was measured at 450 nm using a Spectra Max i3x Multifunctional microplate detection system (Molecular Devices, San Jose, CA).

### 5-Ethynyl-2′-deoxyuridine (EdU) staining

Cells were incubated with DMEM medium containing 50 μM EdU (RiboBio) at 37 °C with 5% CO_2_ for 2 h. Cells were washed twice with PBS, fixed with 50 μL 4% paraformaldehyde for 30 min, neutralized with 50 μL 2 mg/mL glycine solution and permeabilized by adding 100 μL 0.5% Triton X-100. After washing with PBS, 100 μL 1 × Apollo dye was added to each well, then cells were incubated at room temperature for 30 min. Next, 100 μL Hoechst 33342 was added and incubated for another 30 min. Images were captured and analyzed on an ImageXpress high-content screening system (Molecular Devices).

### Colony formation assay

Transfected cells were seeded at a density of 600 cells/well into a 35-mm dish and then cultured for 10 days. Cells were washed with PBS once and colonies were fixed with 4% paraformaldehyde for 20 min and stained with 0.1% crystal violet solution for 10 min at room temperature, followed by image capture.

### Transwell migration and invasion assays

After 24 h transfection, cells were digested, washed twice with PBS and resuspended in serum-free DMEM. Transwell chambers for invasion assay were precoated with Matrigel (BD Biosciences, San Jose, CA). Serum-free DMEM (200 μL) containing cells (4 × 10^4^ cells/well for migration assay, 1 × 10^5^ cells/well for invasion assay) was added to the upper chamber. Then 500 μL DMEM medium supplemented with 20% FBS was added to the lower chamber. After 24 h, cells in the upper chamber were removed with cotton swabs and the lower side of the chamber was gently washed twice with PBS, fixed with 4% paraformaldehyde for 20 min, and stained with 0.1% crystal violet for 10 min, and then images were captured by microscope.

### Apoptosis analysis

Apoptosis was determined using a Dead Cell Apoptosis kit (ThermoFisher Scientific). Briefly, cells were digested with EDTA-free trypsin and washed with ice-cold PBS, followed by a 15-min incubation with Alexa Fluor 488 annexin V and PI, then cells were analyzed by a NovoCyte flow cytometer (ACEA Biosciences, Hangzhou, China).

### Prediction of RNA interaction

Target gene prediction of *let-7c-5p* was performed using the ENCORI online program with strict stringency (http://starbase.sysu.edu.cn/index.php). The interaction between *circCORO1C* and miRNA was predicted by seedVicious v1.0 and RegRNA 2.0 (https://seedvicious.essex.ac.uk/predict.html, http://regrna2.mbc.nctu.edu.tw/index.html).

### TCGA data analysis

Transcriptome sequencing data and clinical features of head and neck squamous cell carcinoma (HNSCC) were downloaded from The Cancer Genome Atlas (TCGA) HNSCC cohort (https://portal.gdc.cancer.gov/projects/TCGA-HNSC), followed by expression analysis of *PBX3* and *let-7c-5p* with normalized FPKM and RPM values.

### RNA immunoprecipitation (RIP)

RIP experiments were performed with a Magna RIP RNA-Binding Protein Immunoprecipitation Kit (Millipore, Billerica, MA) according to the manufacturer’s instructions. Briefly, 1 × 10^7^ cells were collected and resuspended in 300 μL RIPA lysis buffer containing protease inhibitor cocktail and RNase inhibitors. The cell lysates (200 μL) were incubated with 5 μg AGO2 antibody (#2897; CST, Danvers, MA) or rabbit IgG and protein A/G magnetic beads at 4 °C overnight with rotation. Immunoprecipitated RNA was purified using a RNeasy MinElute Cleanup kit (Qiagen). The enrichment of *circCORO1C* was evaluated by qPCR.

### Luciferase reporter assay

HEK293T cells were cotransfected with luciferase reporter plasmid and *let-7c-5p* mimics or NC mimics for 48 h. The luciferase activity was measured using a dual luciferase reporter assay system (Promega) on a Spectra Max i3x Multifunctional microplate detection system (Molecular Devices). The luciferase values were normalized and then the relative luciferase activity was calculated.

### Western blotting

Total protein was extracted with RIPA buffer containing protease inhibitor cocktail (ThermoFisher Scientific). The protein concentration was determined using a Coomassie (Bradford) Protein Assay Kit (ThermoFisher Scientific). Equal amounts of total protein (30 μg) were separated by 12% SDS-PAGE and transferred onto PVDF membranes (Millipore, Billerica, MA), followed by blocking with 5% skim milk. The membranes were incubated with antibodies against PBX3 (#12571-1-AP; Proteintech, Wuhan, China), E-cadherin (#3195S; CST, Danvers, MA), N-cadherin (#13116S; CST), Vimentin (#5741S; CST), Slug (#sc-166476; Santa Cruz Biotechnology), or GAPDH (#HC301-02; TransGen Biotech) overnight at 4 °C. Then membranes were washed three times with TBST followed by secondary antibody incubation for 2 h at room temperature. Bands were detected by a chemiluminescence imaging system (SageCreation Science, Beijing, China) with Western Bright ECL HRP substrate (Advansta Inc., San Jose, CA).

### Xenograft tumorigenesis

SPF-grade male BALB/C nude mice (6-8 weeks) were purchased from Beijing Vital River Laboratory Animal Technology Co., Ltd. (Beijing, China) and housed under SPF conditions (TECNIPLAST S.p.A., Italy). A total of 2 × 10^6^ FD-LSC-1 cells were suspended in 200 μL serum-free DMEM and subcutaneously injected into the right flank of each mouse. The volumes of tumors were measured from 7 days after injection. Tumor volume was calculated as follows: V (volume) = (length × width^2^)/2. After 25 days, the mice were killed and the tumors were dissected, weighed, and processed for histological analysis.

### Immunohistochemical (IHC) staining

IHC staining was performed as previously described [3]. In brief, tissues were fixed in 4% (v/v) formaldehyde in PBS, embedded in paraffin, and cut into 3-μm sections. Sections underwent dewaxing, re-hydration, antigen retrieval, and blocking, and then were incubated with antibodies against PBX3, Ki67, E-cadherin, N-cadherin, and Vimentin overnight at 4 °C in a moist chamber, and washed three times with PBST. Sections were incubated with HRP-conjugated secondary antibody (CST) for 15 min at room temperature, washed three times with PBST, and then stained with DAB and hematoxylin. Next, sections were dehydrated and mounted with coverslips.

### Statistical analysis

Statistical analysis was performed using GraphPad Prism 7.0 software (La Jolla, USA). Comparisons between two groups were performed using the two-tailed Student’s *t*-test. Correlations were analyzed by Pearson’s correlation. Kaplan-Meier survival curve and log-rank test were employed to depict the overall survival probability of LSCC patients with different expression levels of *circCORO1C*. Results are presented as mean ± standard deviation (SD). *P* values of < 0.05 were considered statistically significant.

## Results

### *circCORO1C* is frequently upregulated in LSCC and is associated with malignant progression and poor prognosis

**Fig. 1:**
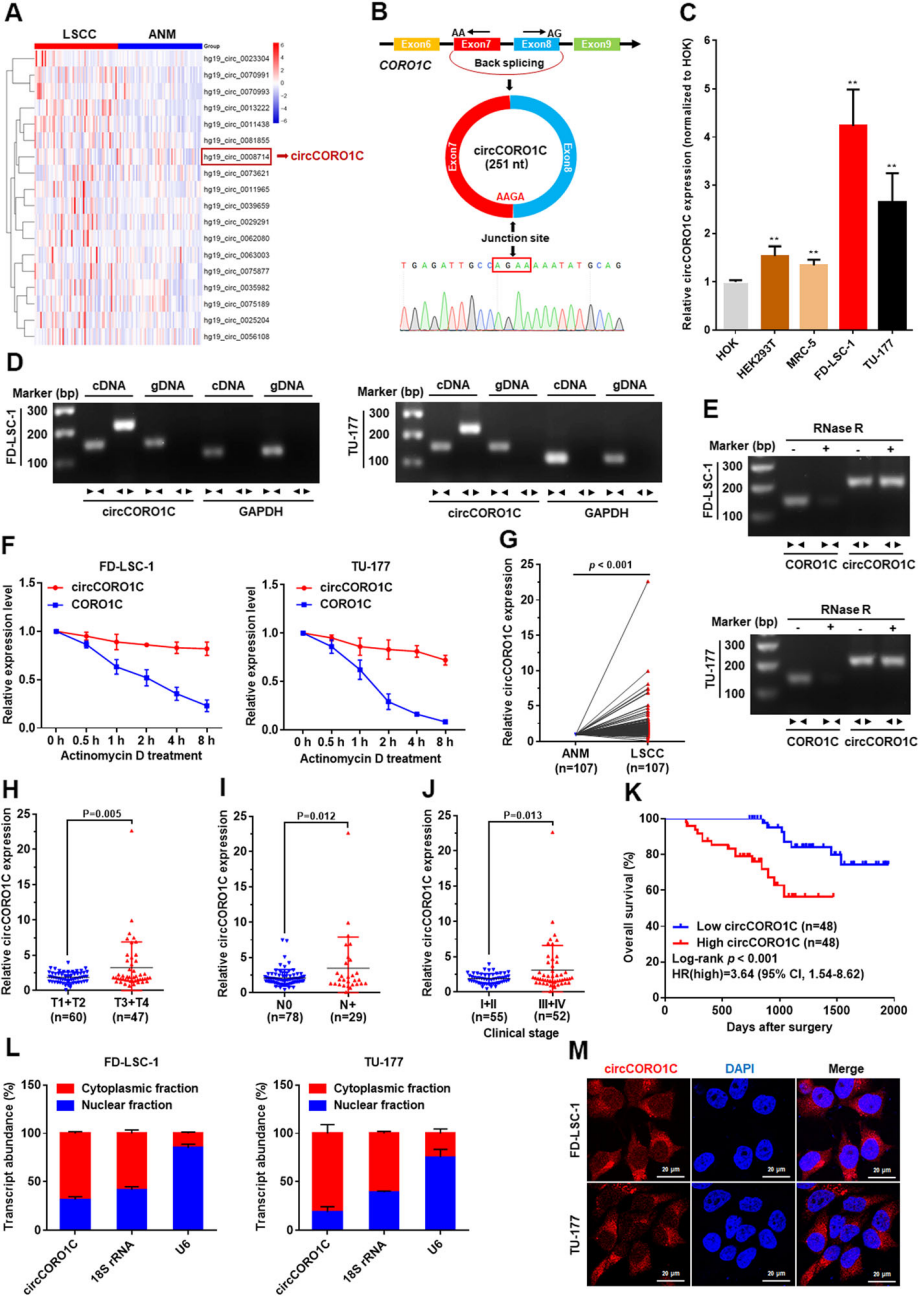
*circCORO1C* is upregulated in LSCC tissues and is associated with poor prognosis. **a** RNA sequencing of 57 pairs of LSCC and matched adjacent normal mucosal (ANM) tissues to screen differentially expressed circRNAs. Heatmap showing circRNAs expressed in all tissues and those upregulated in LSCC tissues. **b** Schematic illustration showed the circularization of *CORO1C* exons 7 and 8 to form *circCORO1C*. The back-splicing junction of *circCORO1C* was verified by RT-PCR and Sanger sequencing. **c** *circCORO1C* expression levels in human LSCC cell lines (FD-LSC-1, TU-177) and normal cell lines (HOK, HEK293T, MRC-5) were determined by qPCR. **d** *circCORO1C* expression in FD-LSC-1 and TU-177 cells verified by RT-PCR. Agarose gel electrophoresis showed that divergent primers amplified *circCORO1C* in cDNA but not genomic DNA (gDNA). *GAPDH* served as a negative control. **e** Validation of *circCORO1C* stability by RNase R treatment and RT-PCR analysis. **f** qPCR analysis of the abundance of *circCORO1C* and linear *CORO1C* in FD-LSC-1 and TU-177 cells treated with actinomycin D at the indicated times. **g** Expression levels of *circCORO1C* in an additional 107 paired LSCC tissues were determined by qPCR. **h–j** Correlation analysis between *circCORO1C* expression levels and clinicopathological parameters of LSCC. Expression level of *circCORO1C* was significantly associated with T stage (**h**), N stage (**i**), and clinical stage (**j**). **k** Kaplan-Meier analysis of the correlation between *circCORO1C* expression and overall survival of 96 patients with LSCC. **l** *circCORO1C* abundance in nuclear and cytoplasmic fractions of FD-LSC-1 and TU-177 cells was evaluated by qPCR. *18S* RNA acted as a positive control of RNA distributed in the cytoplasm, and *U6* RNA acted as a positive control of RNA distributed in the nucleus. **m** Localization of *circCORO1C* in FD-LSC-1 and TU-177 cells was detected by FISH. Nuclei were stained with DAPI (blue) and c*ircCORO1C* probes were labeled with Cy3 (red). The error bars (**c**, **f** and **l**) represent SD of three independent experiments. **P < 0.001

We performed RNA sequencing in 57 pairs of LSCC and matched ANM tissues. Differential expression screening showed that 410 circRNAs were upregulated in LSCC tissues (Additional file 1: Table S3), in which 18 circRNAs were detected in all sequenced tissues (Fig. 1a). Expression of the 18 circRNAs was verified by RT-PCR and Sanger sequencing, and 12 circRNAs were validated successfully (Additional file 2: Figure S1a and b). Next, we screened circRNAs that affect LSCC proliferation by siRNA-mediated knockdown and high-content screening. We found that knockdown of circRNA *hg19_circ_0008714* significantly inhibited LSCC cell proliferation (Additional file 2: Figure S1c). Hence, we focused on this circRNA in this study. Sequence analysis revealed that *hg19_circ_0008714* was formed by back-splicing of exons 7 and 8 of the Coronin-like actin-binding protein 1C gene (*CORO1C*) and was therefore named *circCORO1C* (Fig. 1b). RT-PCR and Sanger sequencing were performed to verify the expression and head-to-tail splicing of *circCORO1C* in LSCC (Fig. 1b). Moreover, we compared the expression levels of *circCORO1C* in LSCC cells and normal control cell lines by qPCR. The results showed that the expression levels of *circCORO1C* in LSCC cell lines FD-LSC-1 and TU-177 were significantly higher than those in normal control cell lines HEK293T, HOK, and MRC-5 (Fig. 1c).

Head-to-tail splicing sequences may be produced by mechanisms other than the formation of circRNA, such as trans-splicing and genomic rearrangements [20]. To rule out the possibility of the latter two, convergent primers for linear and special divergent primers for circular RNA were designed. The convergent primers could be amplified when the template contained *CORO1C* linear mRNA or genomic DNA (gDNA), while the divergent primers could only be specifically amplified in the presence of *circCORO1C*. cDNA and gDNA from FD-LSC-1 and TU-177 cells, respectively, were used as PCR templates. Nucleic acid electrophoresis results indicated that the divergent primers could amplify *circCORO1C* only in cDNA, but no products were detected in the gDNA (Fig. 1d). High stability is a crucial feature of circRNA. To confirm the stability of *circCORO1C*, RNase R was used to pretreat the RNA; the results demonstrated that linear *CORO1C* mRNA was significantly reduced after RNase R treatment, while *circCORO1C* was resistant to RNase R (Fig. 1e). Moreover, we further compared the half-life of the circular form and linear *CORO1C* through treatment with RNA transcription inhibitor actinomycin D and qPCR assay, and found that *circCORO1C* had a significantly longer half-life than the linear *CORO1C* (Fig. 1f)*.* These data confirmed essential features of *circCORO1C.*

To investigate the correlation between *circCORO1C* levels and LSCC, we detected the expression of *circCORO1C* in 107 pairs of LSCC and ANM tissues by qPCR. The relative abundance of *circCORO1C* in LSCC tissues was significantly higher than that in ANM tissues (Fig. 1g). Moreover, the expression level of *circCORO1C* was significantly correlated with T stage, N stage, and clinical stage. Patients in the advanced stage and those with cervical lymph node metastasis had high expression levels of *circCORO1C* (Fig. 1h–j). Importantly, Kaplan-Meier analysis revealed that LSCC patients with high *circCORO1C* levels had poor overall survival (Fig. 1k).

Next, we investigated the location of *circCORO1C* in cells by nuclear and cytoplasmic RNA extraction and qPCR. The results showed that *circCORO1C* was mainly localized to the cytoplasm (Fig. 1l). FISH further confirmed that *circCORO1C* was mainly localized to the cytoplasm (Fig. 1m). These results indicated that *circCORO1C* upregulation is common in LSCC and may have important functions in the progression of LSCC.

### *circCORO1C* promotes the proliferation, migration, and invasion of LSCC cells

**Fig. 2:**
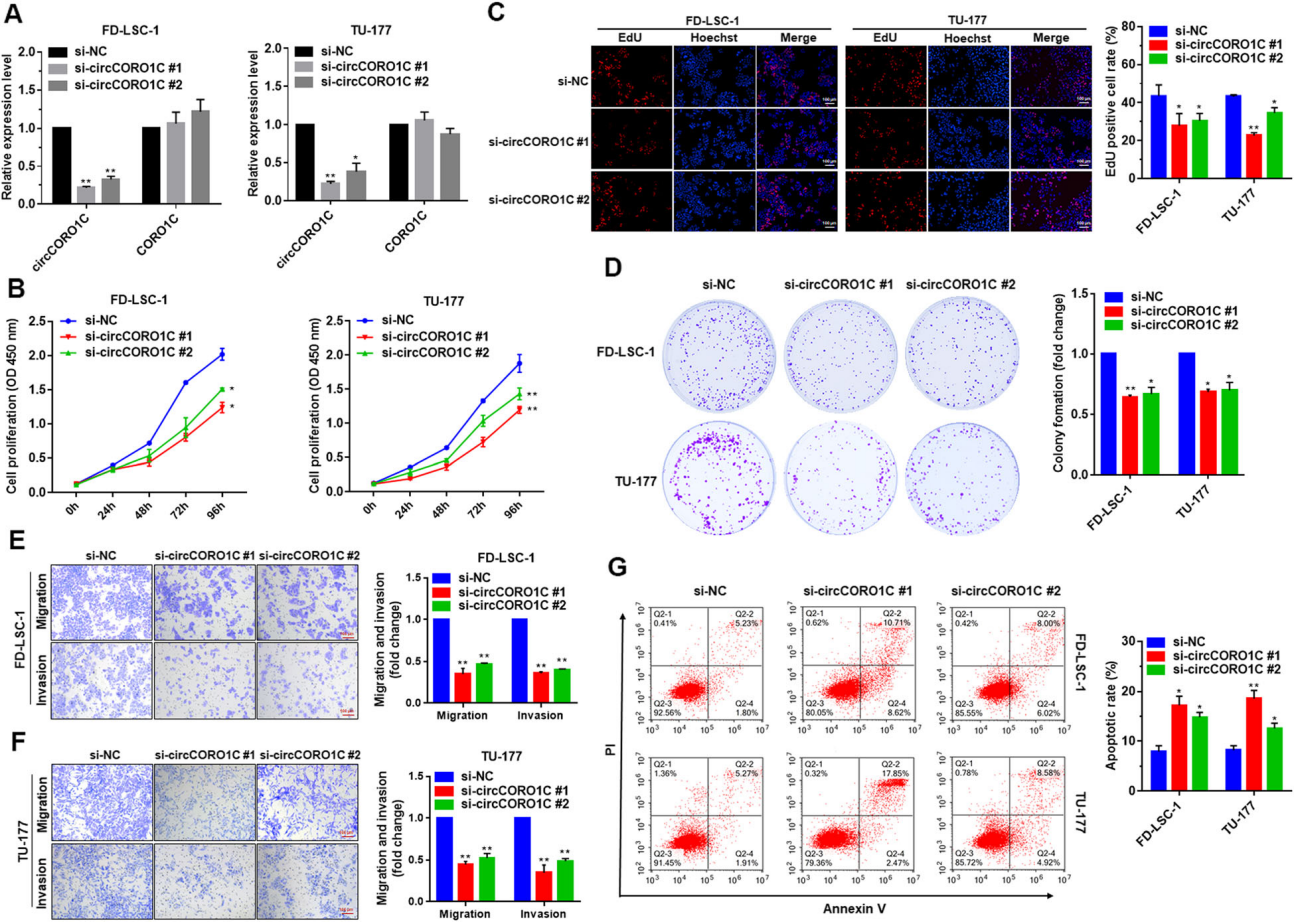
circCORO1C promotes the proliferation, migration, and invasion of LSCC cells. **a** *circCORO1C* siRNA was transfected into FD-LSC-1 and TU-177 cells, and the expression levels of *cicCORO1C* and *CORO1C* were detected by qPCR. **b** FD-LSC-1 and TU-177 cells were transfected with *circCORO1C* siRNAs. Cell proliferation capacity was detected at the indicated time points by CCK8 assays. **c** FD-LSC-1 and TU-177 cells were transfected with *circCORO1C* siRNAs, and the changes in cell proliferation were determined by EdU staining. **d** *circCORO1C* knockdown inhibited colony formation of both FD-LSC-1 and TU-177 cells. **e & f** Knockdown of *circCORO1C* inhibited the migration and invasion of FD-LSC-1 (**e**) and TU-177 (**f**) cells as determined by Transwell migration and invasion assays. **g** FD-LSC-1 and TU-177 cells were transfected with *circCORO1C* siRNAs. Cells were stained with Annexin V-FITC and PI, and the percentage of apoptotic cells was detected by flow cytometry. Data are presented as the mean ± SD of three independent experiments. *P < 0.05; **P < 0.001

To investigate the functions of *circCORO1C* in LSCC cells, we designed and synthesized two siRNAs that specifically targeted the back-splicing region of *circCORO1C*. After LSCC cell lines FD-LSC-1 and TU-177 were transfected with siRNA, qPCR was performed to evaluate the knockdown efficiency. The results showed that both siRNAs could significantly reduce *circCORO1C*, while the level of linear *CORO1C* was not significantly changed, and siRNA #1 had the highest knockdown efficiency (Fig. 2a). We also tested cell viability by CCK-8 assay, and found that knockdown of *circCORO1C* significantly inhibited the viability of LSCC cells (Fig. 2b). EdU staining experiments confirmed that knockdown of *circCORO1C* inhibited the proliferation of LSCC cells (Fig. 2c). Colony formation experiments showed that knockdown of *circCORO1C* significantly inhibited the colony formation of FD-LSC-1 and TU-177 cells (Fig. 2d).

Furthermore, we investigated the effects of *circCORO1C* on the migration and invasion of LSCC cells by Transwell assays. Knockdown of *circCORO1C* significantly decreased cell migration and invasion (Fig. 2e and f). We further investigated the effect of *circCORO1C* on apoptosis, and found that knockdown of *circCORO1C* promoted apoptosis in LSCC cells (Fig. 2g). Taken together, these findings suggested that *circCORO1C* has an oncogenic role in LSCC, and si-*circCORO1C* #1 had the strongest effect on the cell functions, which was consistent with the knockdown efficiency. Thus, in subsequent studies, we performed experiments using si-*circCORO1C* #1.

### *circCORO1C* acts as a miRNA sponge of *let-7c-5p* in LSCC cells

**Fig. 3:**
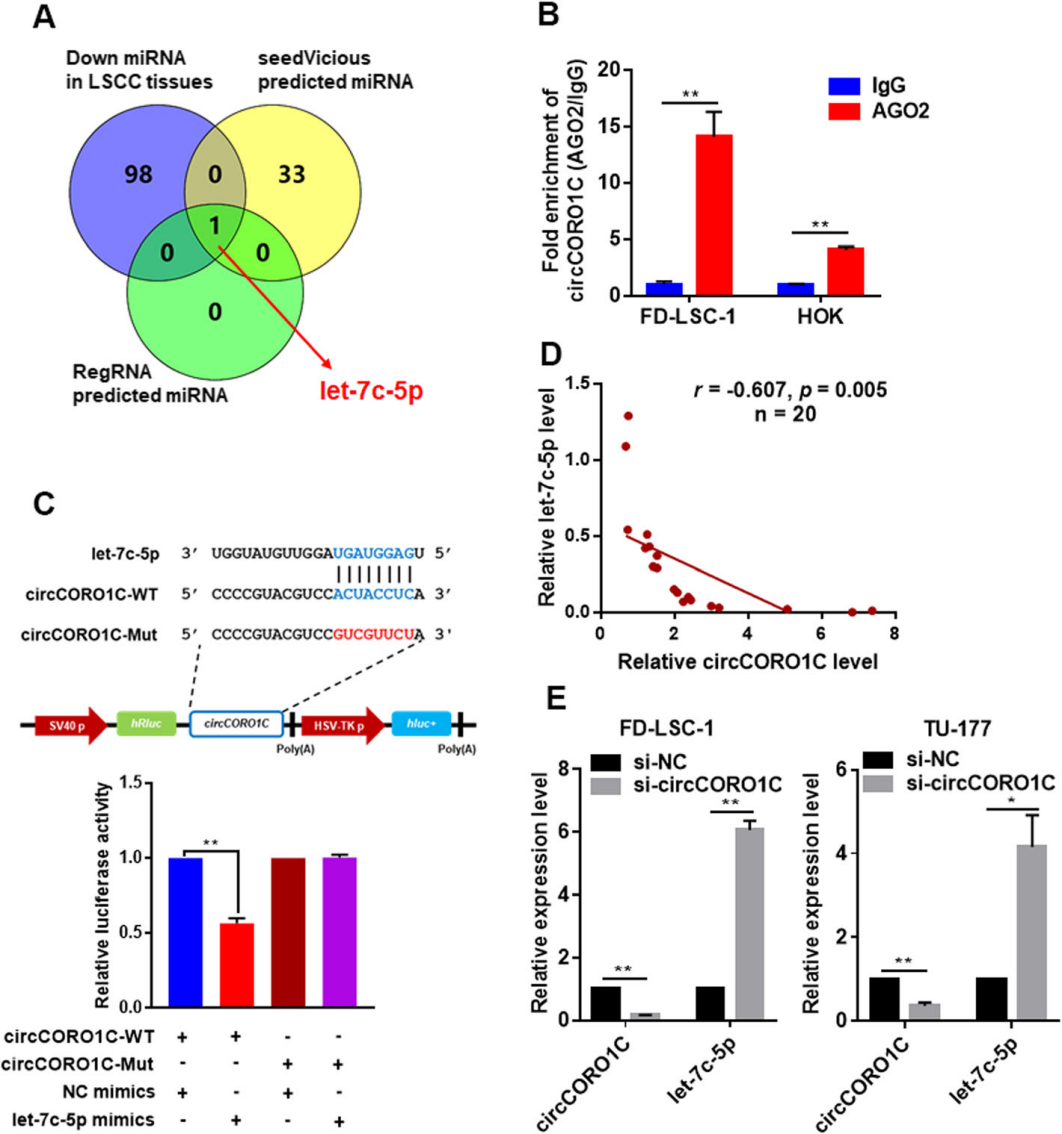
*circCORO1C* acted as a sponge for miRNA *let-7c-5p* in LSCC cells. **a** Combined analysis of bioinformatics prediction and LSCC tissue RNA sequencing data to screen for *circCORO1C*-binding miRNAs. **b** RIP assays were performed using AGO2 antibody in FD-LSC-1 and HOK cells, then the enrichment of *circCORO1C* was detected by qPCR. **c** HEK293T cells were co-transfected with *let-7c-5p* mimics and wild-type or mutant *circCORO1C* luciferase reporter vector, and luciferase reporter activity was detected. **d** Correlation analysis of *circCORO1C* and *let-7c-5p* RNA levels in 20 pairs of LSCC tissues. Expression of *circCORO1C* and *let-7c-5p* in 20 cases of LSCC and matched ANM tissues was determined by qPCR, and the relative expression of *circCORO1C* and *let-7c-5p* was normalized to ANM. **e** Expression levels of *circCORO1C* and *let-7c-5p* in FD-LSC-1 and TU-177 cells transfected with *circCORO1C* siRNAs were evaluated by qPCR. Data are presented as the means ± SD of three independent experiments. *P < 0.05; **P < 0.001

Studies have showed that circRNAs can function as miRNA sponges to competitively bind to miRNA, thus abrogating the inhibitory effect of miRNA on downstream target genes. Since *circCORO1C* is distributed in the cytoplasm, we studied whether it could function as a miRNA sponge in LSCC cells. We used the online tools RegRNA and seedVicious to predict the *circCORO1C*-binding miRNA (Additional file 1: Table S7), and intersected the data with miRNAs that were found to be downregulated in RNA sequencing of our 57 pairs of LSCC and ANM tissues. Notably, *let-7c-5p* was the only one common miRNA in these three datasets (Fig. 3a).

As one of the critical components of RNA-induced silencing complex (RISC), Argonaute 2 (AGO2) is the major protein that mediates the interaction between circRNA and target miRNAs [21]. To demonstrate that *circCORO1C* functions as a miRNA sponge, we performed RIP assay using AGO2 antibody in FD-LSC-1 and HOK cells. The results showed that *circCORO1C* was pulled down with AGO2 and was less enriched in HOK cells compared with FD-LSC-1 cells, which consistent with the differential *circCORO1C* levels of these two cell lines (Fig. 3b). In addition, the results of RIP in *let-7c-5p*-transfected FD-LSC-1 cells indicated that *circCORO1C* was specifically enriched by AGO2 antibody (Additional file 2: Figure S2). Moreover, we constructed luciferase reporter vectors for wild-type *circCORO1C* and *let-7c-5p* binding site mutant *circCORO1C* and co-transfected HEK293T cells with *let-7c-5p* mimics or NC mimics. The results showed that the luciferase activity in the wild-type group co-transfected with *let-7c-5p* mimics was significantly reduced, while the luciferase activity in the binding site mutant group was not significantly changed (Fig. 3c). Furthermore, the expression correlation between *circCORO1C* and *let-7c-5p* was analyzed by qPCR in 20 pairs of LSCC tissues, revealing that *let-7c-5p* levels were negatively correlated with *circCORO1C* levels in LSCC tissues (Fig. 3d). In addition, we found that expression of *let-7c-5p* in FD-LSC-1 and TU-177 cells was increased significantly after *circCORO1C* knockdown (Fig. 3e). Collectively, these results indicated that *circCORO1C* functions as a miRNA sponge to directly interact with *let-7c-5p* in LSCC cells.

### *let-7c-5p* is downregulated in LSCC tissues and inhibits the malignant phenotype of LSCC cells

**Fig. 4:**
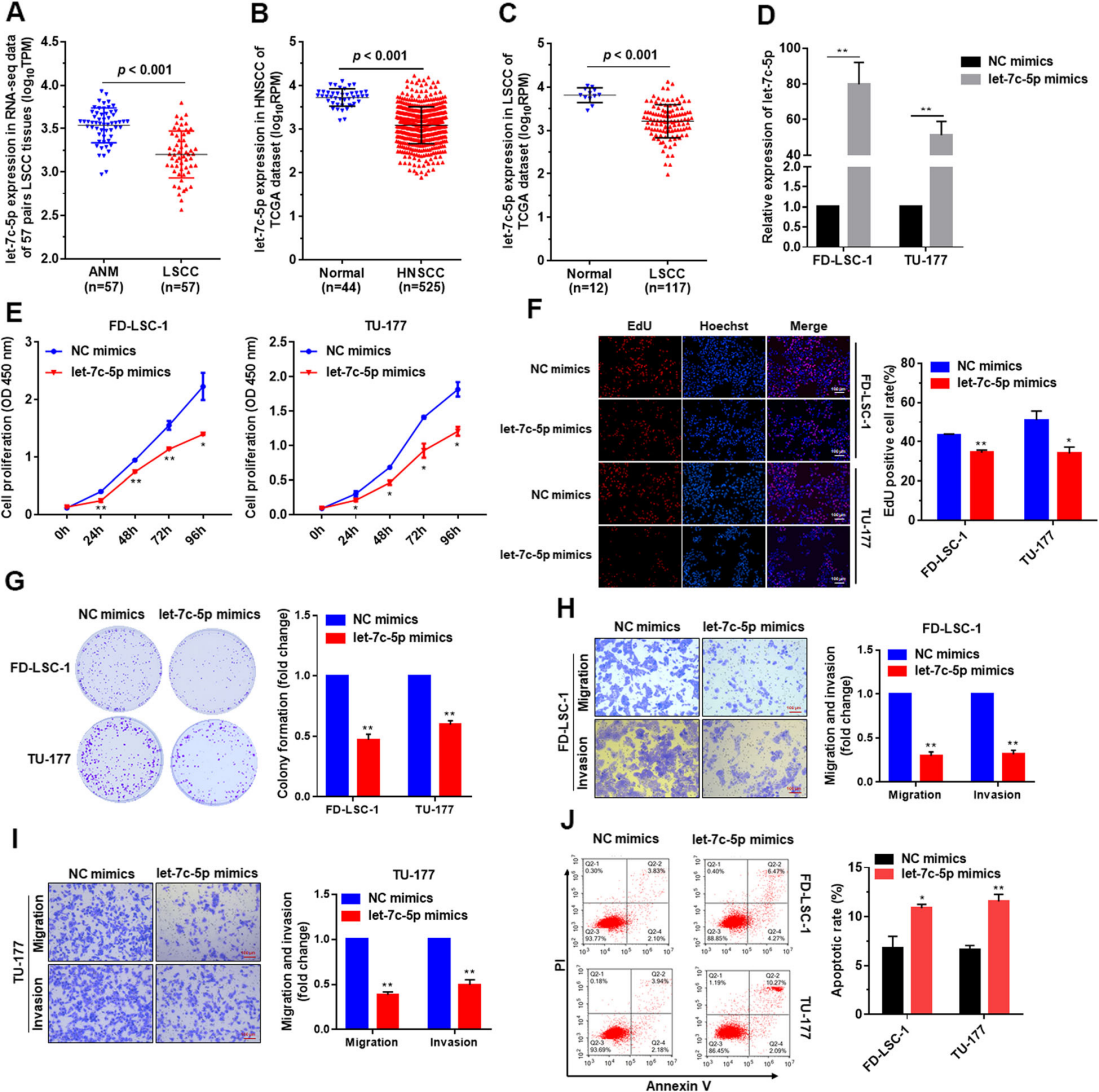
*let-7c-5p* inhibited the proliferation, migration, and invasion of LSCC cells. **a** Expression of *let-7c-5p* in 57 LSCC tissues and matched ANM tissues was analyzed using RNA sequencing data. **b & c** Analysis of *let-7c-5p* expression in HNSCC (**b**) and LSCC (**c**) tissues using transcriptomic sequencing data from TCGA database. **d** FD-LSC-1 and TU-177 cells were transfected with *let-7c-5p* mimics or NC mimics, then *let-7c-5p* expression was determined by qPCR. **e** FD-LSC-1 and TU-177 cells were transfected with *let-7c-5p* mimics or NC mimics. Cell proliferation was detected by CCK8 assay. **f** FD-LSC-1 and TU-177 cells were transfected with *let-7c-5p* mimics or NC mimics, and cell proliferation was assessed by EdU staining. **g** Proliferative capacity of FD-LSC-1 and TU-177 cells transfected with *let-7c-5p* mimics or NC mimics was evaluated by colony formation assay. **h & i**. Effect of *let-7c-5p* on the migration and invasion of FD-LSC-1 (**h**) and TU-177 (**i**) cells was assessed by Transwell migration and invasion assays. **j** FD-LSC-1 and TU-177 cells were transfected with *let-7c-5p* mimics or NC mimics. Cells were stained with Annexin V-FITC and PI, and the percentage of apoptotic cells was detected by flow cytometry. Data are presented as the mean ± SD of three independent experiments. *P < 0.05; **P < 0.001

RNA sequencing data indicated that *let-7c-5p* expression levels in LSCC tissues were significantly lower than those in ANM tissues (Fig. 4a). Analysis of TCGA data confirmed that *let-7c-5p* expression was downregulated in HNSCC and LSCC (Fig. 4b and c), indicating that *let-7c-5p* may have important roles in LSCC. Therefore, we investigated the functions of *let-7c-5p* in LSCC cells. FD-LSC-1 and TU-177 cells were transfected with *let-7c-5p* mimics or negative control mimics (NC mimics), then the transfection efficiency was verified by qPCR, which revealed that *let-7c-5p* expression was elevated (Fig. 4d). CCK8 assay and EdU staining indicated that overexpression of *let-7c-5p* inhibited LSCC cell proliferation (Fig. 4e and f). Colony formation experiments found that the colony formation ability of LSCC cells overexpressing *let-7c-5p* was significantly decreased (Fig. 4g), while Transwell assay showed that the migration and invasion of LSCC cells were significantly attenuated after overexpression of *let-7c-5p* (Fig. 4h and i). Apoptosis assay showed that *let-7c-5p* overexpression promoted the apoptosis of LSCC cells (Fig. 4j). Overall, these data demonstrated that *let-7c-5p* inhibits the proliferation, migration, and invasion of LSCC cells and promotes their apoptosis.

### *let-7c-5p* reversed the tumor-promoting effects of *circCORO1C* in LSCC cells

**Fig. 5:**
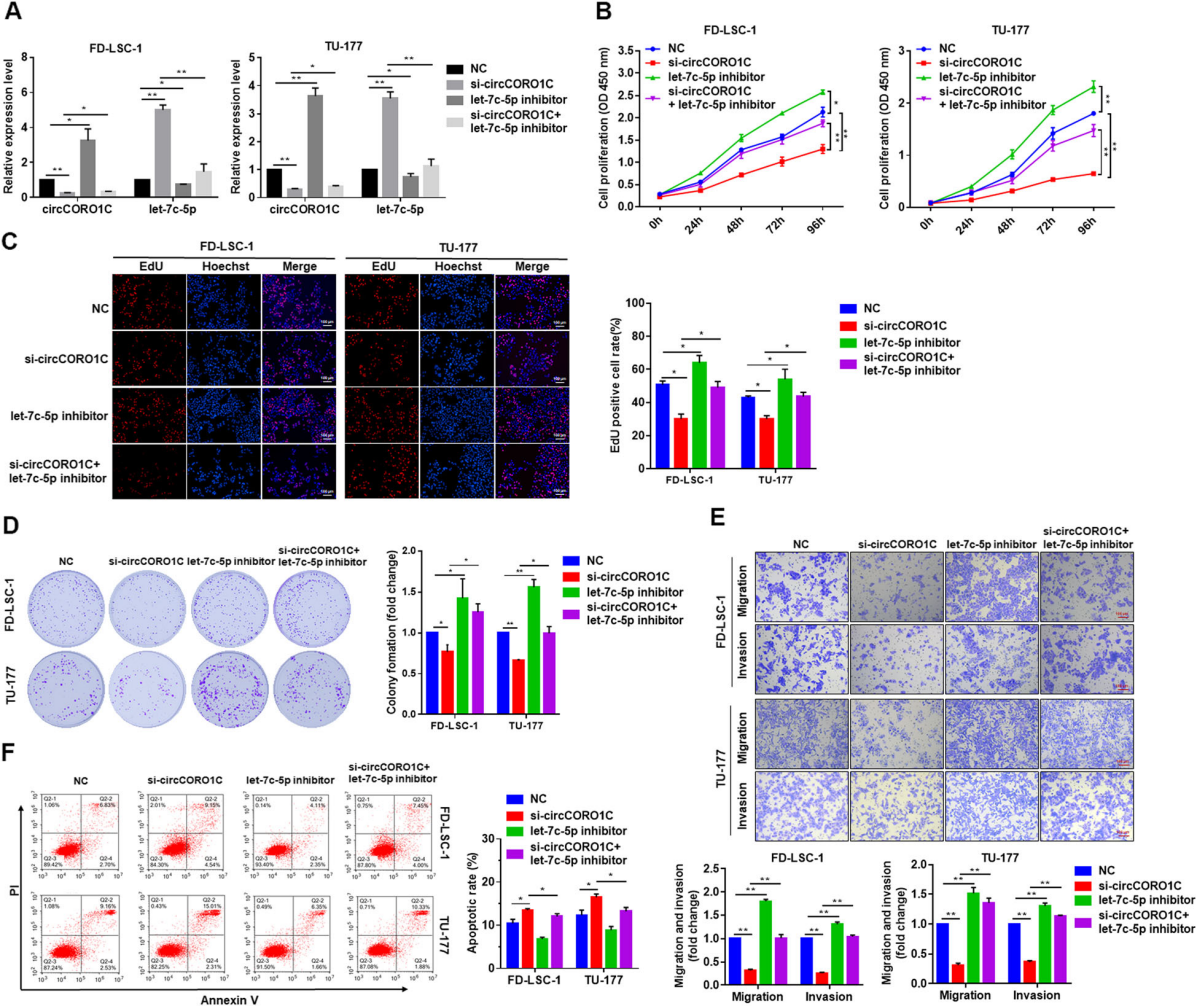
*let-7c-5p* reversed the tumor-promoting effect of *circCORO1C* in LSCC cells. **a** FD-LSC-1 and TU-177 cells were transfected with si-*circCORO1C* or co-transfected with si-*circCORO1C* and *let-7c-5p* inhibitor. *CircCORO1C* and *let-7c-5p* expression was detected by qPCR. **b** FD-LSC-1 and TU-177 cells were transfected with si-*circCORO1C* or co-transfected with si-*circCORO1C* and *let-7c-5p* inhibitor. Cell proliferation was determined by CCK8 assay. **c** Effects of si-*circCORO1C* and *let-7c-5p* inhibitor on the proliferation of FD-LSC-1 and TU-177 cells were evaluated by EdU staining. **d** Colony formation assays were performed to evaluate the proliferative ability of FD-LSC-1 and TU-177 cells transfected with si-*circCORO1C* or co-transfected with si-*circCORO1C* and *let-7c-5p* inhibitor. **e** Effects of si-*circCORO1C* and *let-7c-5p* inhibitor on the migration and invasion of FD-LSC-1 and TU-177 cells were evaluated by Transwell migration and invasion assays. **f** FD-LSC-1 and TU-177 cells were transfected with si-*circCORO1C* or co-transfected with si-*circCORO1C* and *let-7c-5p* inhibitor*.* Cells were stained with Annexin V-FITC and PI, and the percentage of apoptotic cells was detected by flow cytometry. Data are presented as the means ± SD of three independent experiments. *P < 0.05; **P < 0.001

To identify whether *circCORO1C* promoted LSCC cell proliferation, migration, and invasion by interacting with *let-7c-5p*, we conducted a rescue experiment. FD-LSC-1 and TU-177 cells were cotransfected with si-*circCORO1C* and *let-7c-5p* inhibitor (Fig. 5a), and then CCK-8, EdU staining, and colony formation assays were conducted. The results showed that when *let-7c-5p* function was inhibited with miRNA inhibitor, the proliferation and colony formation ability of LSCC cells was significantly enhanced (Fig. 5b–d). Notably, transfection with *let-7c-5p* inhibitor could reverse the decreased cell viability caused by si-*circCORO1C* (Fig. 5b–d). Transwell assays showed that *let-7c-5p* inhibition reversed the reduction in the migration and invasion of FD-LSC-1 and TU-177 cells caused by *circCORO1C* knockdown (Fig. 5e). Compared with cells transfected with si-*circCORO1C* alone, co-transfection of LSCC cells with si-*circCORO1C* and *let-7c-5p* inhibitor significantly reduced apoptosis (Fig. 5f). Taken together, these results indicated that *circCORO1C* promoted the malignant progression of LSCC cells mainly by abolishing the anti-tumor effect of *let-7c-5p*.

### *PBX3* is a direct target of *let-7c-5p* and functions as driver gene in LSCC

**Fig. 6:**
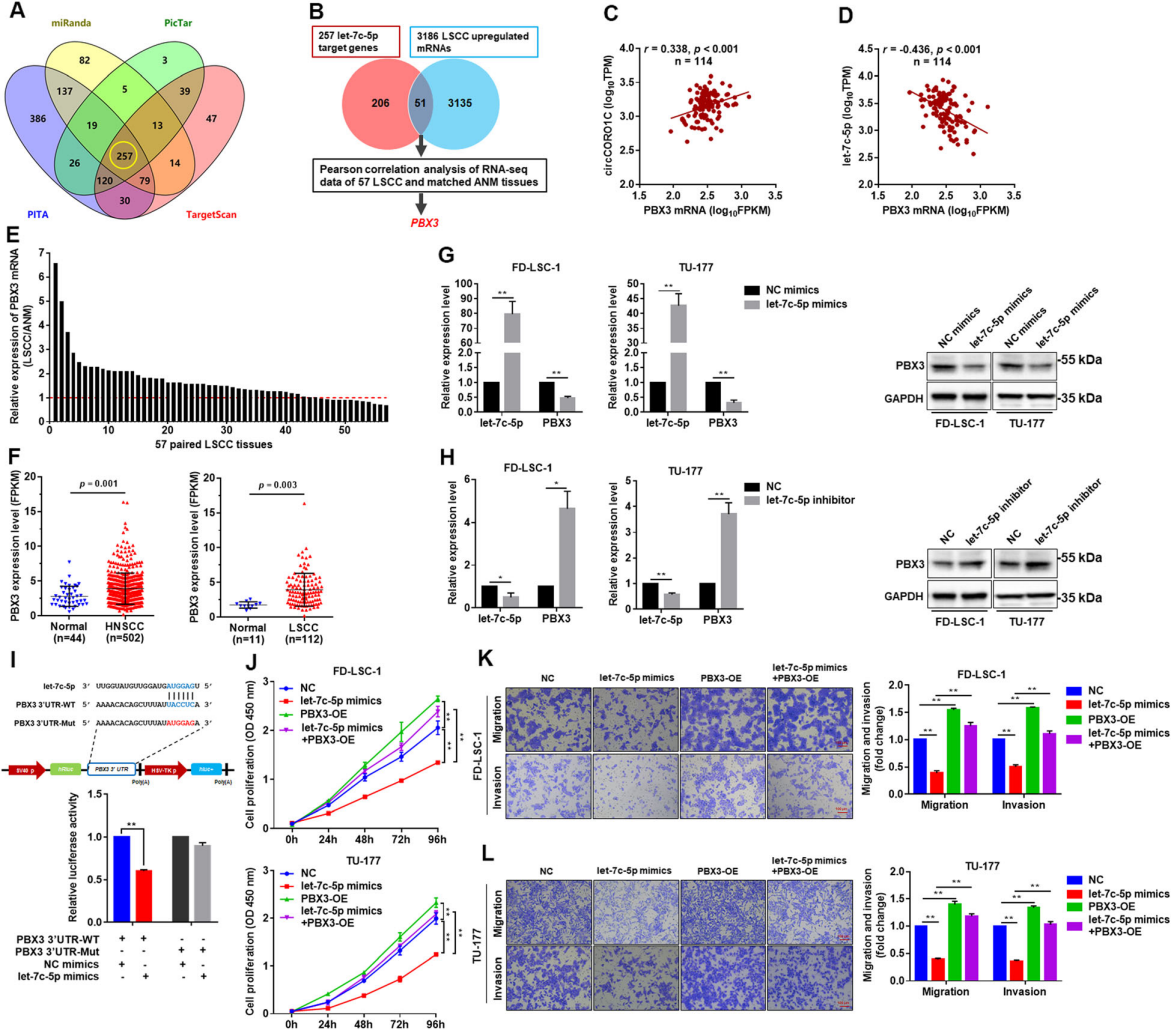
*PBX3* is a direct target gene of *let-7c-5p*, which acted as an oncogene in LSCC cells. **a** Venn analysis of the target genes of *let-7c-5p* predicted by miRanda, PicTar, PITA, and TargetScan. **b** Integrated analysis of bioinformatics-predicted target genes and RNA sequencing data of 57 pairs of LSCC tissues was performed to screen for *let-7c-5p* target genes. **c & d** Correlation analysis between *circCORO1C* (**c**) or *let-7c-5p* (**d**) and *PBX3* expression using RNA sequencing data of 57 pairs of LSCC tissues and matched ANM tissues. **e** *PBX3* expression in RNA sequencing data of 57 pairs of LSCC tissues and matched ANM tissues. The expression levels of *PBX3* in each LSCC tissue were normalized to corresponding matched ANM tissue. **f** Analysis of *PBX3* expression in HNSCC and LSCC tissues using transcriptome sequencing data from TCGA database. **g & h** FD-LSC-1 and TU-177 cells were transfected with *let-7c-5p* mimics (**g**), *let-7c-5p* inhibitor (**h**) or NC, and PBX3 expression was detected by qPCR and western blotting. **i** HEK293T cells were co-transfected with *let-7c-5p* mimics and wild-type or mutant *PBX3* 3′ UTR reporter plasmids, and luciferase reporter assays were performed to evaluate the effect of *let-7c-5p* on luciferase activity. **j** FD-LSC-1 and TU-177 cells were transfected with *let-7c-5p* mimics or co-transfected with *let-7c-5p* mimics and *PBX3* overexpression plasmids, and CCK8 assay was performed to detect cell proliferation. **k & l** FD-LSC-1 (**k**) and TU-177 (**l**) cells were transfected with *let-7c-5p* mimics or co-transfected with *let-7c-5p* mimics and *PBX3* overexpression plasmids. Changes in cell migration and invasion capacity were evaluated by Transwell assays. Data are presented as the means ± SD of three independent experiments. *P < 0.05; **P < 0.001

According to ceRNA theory, *circCORO1C* is positively correlated with the expression of downstream target genes, while the target gene is negatively correlated with *let-7c-5p* expression. We predicted the possible *let-7c-5p* target genes by miRanda, PicTar, PITA, and TargetScan, and 257 genes intersected by these four programs were obtained (Fig. 6a; Additional file 1: Table S8). Then we intersected these 257 genes with the mRNAs that were found to be upregulated in LSCC tissues upon RNA sequencing, and 51 intersected genes were obtained (Fig. 6b; Additional file 1: Table S9). Next, we analyzed the expression correlation of *circCORO1C* and *let-7c-5p* with the 51 genes using RNA sequencing data of 57 pairs of LSCC samples. Pearson correlation analysis indicated that *circCORO1C* was positively correlated with *PBX3*, while *let-7c-5p* was negatively correlated with *PBX3* in LSCC and ANM tissues (Fig. 6c and d). RNA sequencing data showed that *PBX3* was upregulated in 73.7% (42/57) of LSCC tissues (Fig. 6ec and d). Moreover, analysis of the transcriptomic data of TCGA database found that *PBX3* was upregulated in both HNSCC and LSCC (Fig. 6f). In addition, overexpression of *let-7c-5p* significantly decreased the expression of PBX3 mRNA and protein (Fig. 6g), while downregulation of *let-7c-5p* remarkably increased it in FD-LSC-1 and TU-177 cells (Fig. 6h).

To demonstrate that *let-7c-5p* interacts directly with the 3′ UTR of *PBX3*, we constructed wild-type (WT) *PBX3* 3′ UTR and *let-7c-5p* binding-site mutant (Mut) luciferase reporter plasmids. The wild-type and mutant reporter vectors were co-transfected with *let-7c-5p* mimics in cells. Luciferase reporter assays showed that *let-7c-5p* mimics significantly decrease the luciferase activity of WT, while the luciferase activity of the Mut group was not significantly changed (Fig. 6i), indicating that *let-7c-5p* suppresses PBX3 expression by directly binding to the 3′ UTR of *PBX3* mRNA.

Subsequently, we investigated the functions of PBX3 in LSCC cells. Overexpression of PBX3 promoted the proliferation, migration, and invasion of LSCC cells (Fig. 6j–l). Notably, we observed that overexpression of PBX3 counteracted the inhibitory effects of *let-7c-5p* on LSCC cell proliferation, migration, and invasion (Fig. 6j–l). Collectively, these findings suggested that *PBX3* is a driver gene and a direct target of *let-7c-5p* in LSCC.

### *circCORO1C* facilitates the malignant progression of LSCC cells by targeting PBX3

**Fig. 7:**
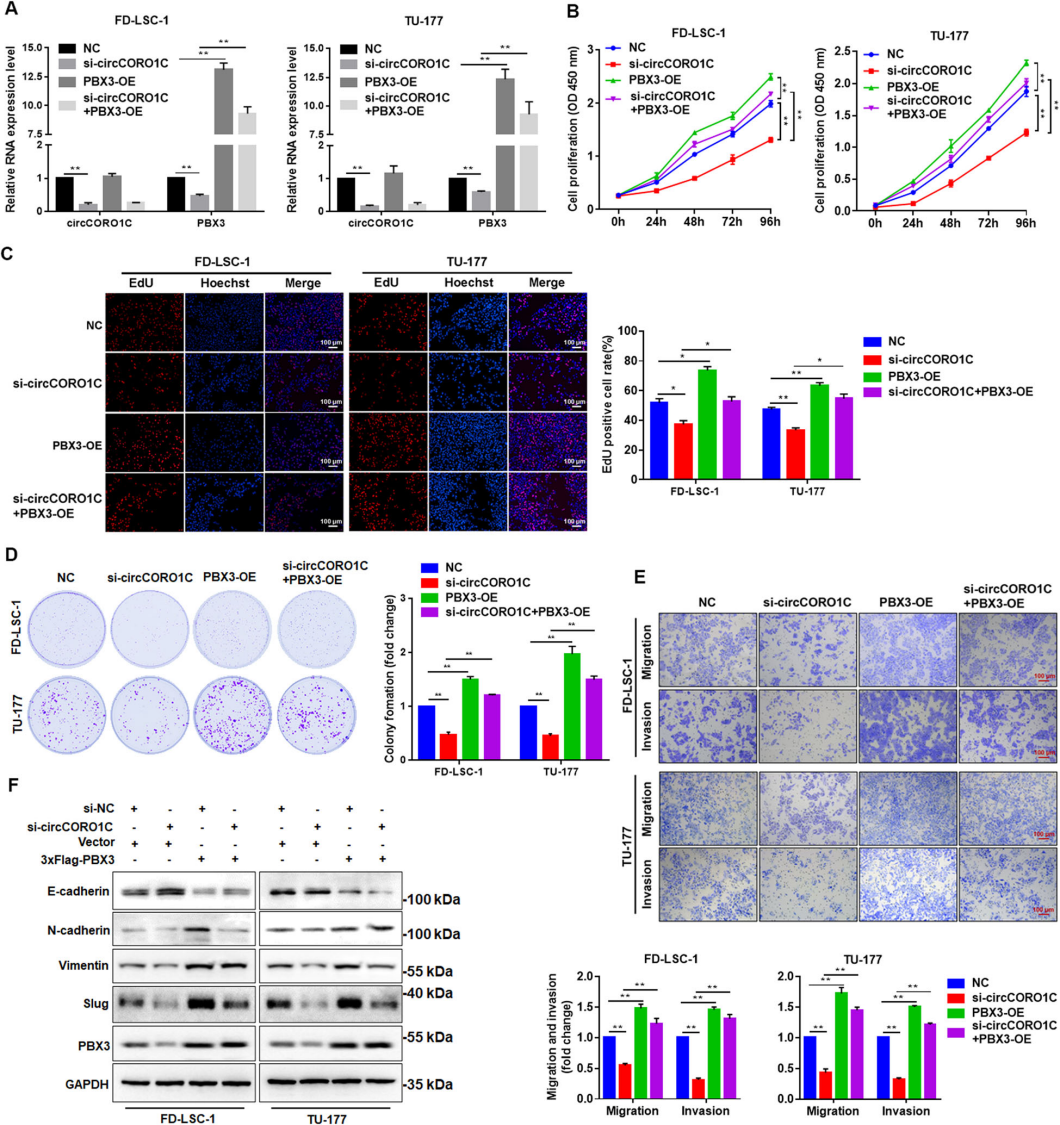
*CircCORO1C* contributed to the malignant phenotype of LSCC cells through regulating the expression of *PBX3*. **a** FD-LSC-1 and TU-177 cells were transfected with si-*circCORO1C* or co-transfected with si-*circCORO1C* and *PBX3* overexpression plasmids. *PBX3* expression was detected by qPCR. **b & c** FD-LSC-1 and TU-177 cells were transfected with si-*circCORO1C* or co-transfected with si-*circCORO1C* and *PBX3* overexpression plasmids. Cell proliferation was determined by CCK8 assay (**b**) and EdU staining (**c**). **d** FD-LSC-1 and TU-177 cells were transfected with si-*circCORO1C* or co-transfected with si-*circCORO1C* and *PBX3* overexpression plasmids. Cell proliferation ability was evaluated by colony formation assay. **e** Effects of *circCORO1C* knockdown and overexpression of *PBX3* on the migration and invasion of FD-LSC-1 and TU-177 cells were determined by Transwell assays. **f** E-cadherin, N-cadherin, Vimentin, and Slug expression in FD-LSC-1 and TU-177 cells with knockdown of *circCORO1C* and overexpression of *PBX3* were detected by western blotting. Data are presented as the means ± SD of three independent experiments. *P < 0.05; **P < 0.001

To investigate whether *circCORO1C* promoted the malignant progression of LSCC cells by regulating the downstream target gene *PBX3*, we simultaneously transfected *PBX3* overexpression plasmid and si-*circCORO1C* into FD-LSC-1 and TU-177 cells (Fig. 7a) and detected changes in the cell phenotypes. CCK-8 assay and EdU staining were performed, and the results showed that overexpression of *PBX3* could inhibit the decrease in cell proliferation caused by *circCORO1C* knockdown (Fig. 7b and c). Consistently, overexpression of *PBX3* rescued the decreased colony formation ability by *circCORO1C* knockdown (Fig. 7d). Furthermore, Transwell assay showed that overexpression of *PBX3* could reverse the decline in cell migration and invasion ability caused by *circCORO1C* knockdown (Fig. 7e). We also detected protein changes in EMT marker genes by western blotting. *circCORO1C* knockdown enhanced the expression of E-cadherin while inhibiting the expression of N-cadherin, Vimentin, and Slug (Fig. 7f), and overexpression of *PBX3* could reverse the regulatory effects of *circCORO1C* on these EMT markers. Compared with the si-*circCORO1C* group, the expression of E-cadherin was reduced, and the expression of N-cadherin, Vimentin, and Slug were increased in the group cotransfected with si-*circCORO1C* and *PBX3* overexpression plasmid (Fig. 7f). These findings indicated that *circCORO1C* promoted the proliferation, migration, and invasion phenotype of LSCC cells by specifically upregulating the expression of the target gene *PBX3* and affecting the EMT process at the same time.

### *circCORO1C* enhances the growth of xenograft tumors of LSCC cells in vivo

**Fig. 8:**
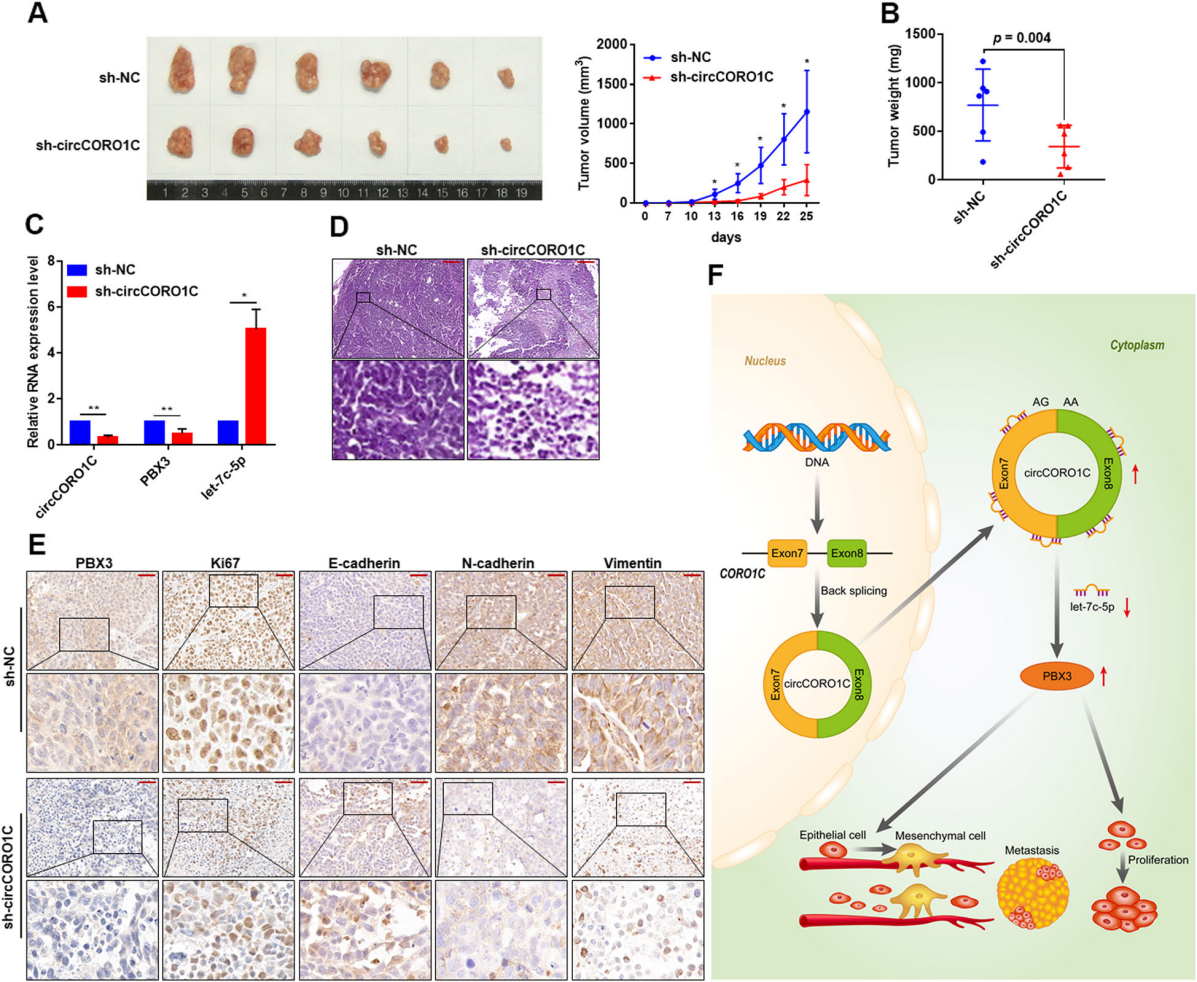
*CircCORO1C* promoted the tumor growth of LSCC cells in vivo. **a** Nude mice were subcutaneously injected with negative control (sh-NC) and shRNA-*circCORO1C* stably transfected FD-LSC-1 cells. After 25 days, tumors were dissected and imaged (left). Starting from day 7 after injection, the tumor volume was measured every 3 days, and the tumor growth curve was plotted (right). **b** Tumor weight was calculated on the day the mice were killed. Data represents mean ± SD (*n* = 6 each group). **c** Expression levels of *circCORO1C, let-7c-5p*, and *PBX3* in xenograft tumors were determined by qPCR. **d** H&E staining revealed the structure of xenograft tumors derived from sh-NC and sh-*circCORO1C* LSCC cells. Scale bar, 200 μm. **e** Changes in PBX3, Ki67, E-cadherin, N-cadherin, and Vimentin expression in xenograft tumors were detected by IHC staining. Scale bar, 20 μm. **f** Schematic illustration of the regulation of LSCC malignant progression by the *circCORO1C*–*let-7c-5p*–*PBX3* axis. *P < 0.05; **P < 0.001

To investigate the regulatory effect of *circCORO1C* on LSCC under in vivo conditions, we constructed a shRNA lentiviral plasmid targeting *circCORO1C* and screened FD-LSC-1 cells following stable knockdown of *circCORO1C* (sh-circCORO1C). Next, we constructed xenograft tumor models of nude mice by subcutaneously injecting stably transfected FD-LSC-1 cells. The xenograft tumors formed by *circCORO1C*-deficient LSCC cells had a significantly smaller volume than those of the control group (sh-NC) (Fig. 8a), and the tumor weight was also significantly lower than the sh-NC group (Fig. 8b). The total RNA of xenograft tumors was extracted, and qPCR was used to detect the expression of *circCORO1C*, *let-7c-5p*, and *PBX3*. The results confirmed decreased *circCORO1C* and *PBX3* expression, while *let-7c-5p* was increased in tumors with *circCORO1C* knockdown (Fig. 8c). Furthermore, hematoxylin and eosin (H&E) staining showed that knockdown of *circCORO1C* remarkably reduced the number of lesions (Fig. 8d). IHC staining demonstrated that the expression of PBX3 and proliferation marker Ki67 was decreased in sh-*circCORO1C* xenograft tumors (Fig. 8e). In addition, the changes in EMT marker E-cadherin, N-cadherin, Vimentin, and Slug expression were determined by IHC. The results revealed that knockdown of *circCORO1C* attenuated the mesenchymal phenotype (Fig. 8e). These data confirmed that *circCORO1C* promoted the malignant progression of LSCC in vivo.

## Discussion

Studies have shown that circRNA has important regulatory effects in a variety of biological processes, especially in the occurrence, development, and metastasis of various malignant tumors [22–24]. circRNA expression profiling revealed a series of differentially expressed circRNAs in LSCC tissues [25, 26], suggesting that circRNA may have important roles in the occurrence and progression of LSCC. In this study, we performed large-scale RNA sequencing of LSCC and matched ANM tissues, and established the circRNA, miRNA, and mRNA expression profiles of LSCC tissues. We identified and verified that *circCORO1C* was highly expressed in LSCC tissues and cells, and its expression levels were correlated with clinicopathological parameters and LSCC patient survival. Loss-of-function experiments demonstrated that *circCORO1C* promoted the proliferation, migration, and invasion of LSCC cells and inhibited their apoptosis. Mechanistic studies showed that *circCORO1C* bound to *let-7c-5p* and attenuated the inhibition of *let-7c-5p* on the target gene *PBX3*, leading to PBX3 accumulation and enhancing the proliferation, migration, and invasion of LSCC cells.

The *CORO1C*-encoded WD repeat protein family member regulates actin-dependent processes through F-actin assembly [27]. Studies have shown that *CORO1C* promotes the metastases of breast cancer and lung squamous cell carcinoma [28, 29]. Cheng et al. reported that *CORO1C* is highly expressed in gastric cancer tissues, and in vitro experiments demonstrated that *CORO1C* promotes the proliferation, migration, and invasion of gastric cancer cells [30]. However, it is unclear whether circRNA is formed by *CORO1C*, and the roles of *CORO1C*-formed circRNA in disease or normal physiological processes have not yet been reported. In this study, RNA sequencing data analysis and experiments demonstrated that *circCORO1C*, which was highly expressed in LSCC tissues, was composed of exons 7 and 8 of *CORO1C*. Treatment with actinomycin D showed that the half-life of *circCORO1C* was significantly longer than that of linear *CORO1C* RNA. RNase R has 3′ to 5′ exoribonuclease activity that digests all linear RNAs except circular RNA structures [31]. When treated with RNase R, there is no significant change in *circCORO1C* level, proving that it has high stability as previously reported circRNA [32, 33]. Importantly, we found that the high expression of *circCORO1C* was positively correlated with advanced T stage, cervical lymph node metastasis, and clinical stage of LSCC, as well as poor prognosis in patients with LSCC, suggesting that *circCORO1C* may exert an important regulatory effect on the occurrence and development of LSCC.

RNA sequencing and bioinformatics analysis indicated that circRNA has an important regulatory effect in the occurrence and development of head and neck tumors [34]. Experimental studies further demonstrated that *circHIPK3* promotes cell proliferation and invasion in nasopharyngeal carcinoma [35], while *Hsa_circ_0005379* inhibits the cell migration, invasion, proliferation, and in vivo tumorigenesis of oral squamous cell carcinoma [36]. CircRNAs *CDR1as* and *hsa_circ_0023028* promote the proliferation, migration, and invasion of LSCC cells [37, 38]. Moreover, our previous studies found that the circRNA *hg19_circ_0005033*, which is highly expressed in LSCC stem cells, promotes proliferation, migration, invasion, and chemotherapy resistance [19]. There are very few LSCC cell lines available, among which FD-LSC-1 and TU-177 are well-characterized [18, 39]. Our data showed that expression of *circCORO1C* in FD-LSC-1 and TU-177 cells was higher than that in normal control cell lines. Therefore, we used these two cell lines to investigate the role of *circCORO1C* in LSCC cells. Consistent results showed that knockdown of *circCORO1C* inhibited cell proliferation, migration, invasion, and promoted apoptosis of LSCC, indicating that *circCORO1C* acts as an important oncogene to promote the malignant progression of LSCC.

Transcripts with the same miRNA binding site, such as circRNA, mRNA, and lncRNA, regulate the expression of each other by competitively binding miRNAs. These molecules form a complex and precise post-transcriptional regulatory network, namely the ceRNA network [40]. As an important member of the ceRNA network, circRNA is involved in the formation of the circRNA–miRNA–mRNA axis, which has regulatory functions in a variety of diseases and is the most reported mechanism of action of circRNA [41–43]. In this study, we found that *circCORO1C* was localized to the cytoplasm, suggesting that it functions as a ceRNA [44]. *let-7c-5p* has been demonstrated to have anti-tumor effects in malignant tumors including non-small cell lung cancer and liver cancer [45, 46]. The combined bioinformatics prediction and transcriptomic analysis showed that *let-7c-5p* may bind to *circCORO1C*. We further demonstrated that *let-7c-5p* expression levels in LSCC were significantly lower than that in adjacent normal tissues, and overexpression of *let-7c-5p* inhibited cell proliferation, migration, and invasion in LSCC. The luciferase reporter assay and AGO2 RIP experiments demonstrated that *let-7c-5p* bound to *circCORO1C*, while rescue experiments revealed that inhibition of *let-7c-5p* reversed the inhibitory effect of knockdown of *circCORO1C* on LSCC malignant phenotypes. These findings indicated that *circCORO1C* sponged *let-7c-5p* to exert tumor-promoting functions in LSCC cells.

PBX3 is highly expressed in a variety of cancer tissues, such as prostate and cervical cancer [14, 16]. Han et al. demonstrated that PBX3 expression is a critical determinant for maintaining the characteristics of tumor-initiating cells in hepatocellular carcinoma [17]. In this study, we found that PBX3 expression was upregulated in LSCC tissues, and functional studies indicated that PBX3 promoted cell proliferation, migration, and invasion in LSCC. Our data revealed that *PBX3* was a direct target of *let-7c-5p*, and *circCORO1C* competitively bound to *let-7c-5p* and relieved the inhibitory effect of *let-7c-5p* on *PBX3* expression, thereby upregulating *PBX3* expression. We further confirmed that *circCORO1C* promoted the malignant progression of LSCC cells by upregulating *PBX3*. EMT is the basis of tumor cell migration and invasion [47, 48], and PBX3 is an essential regulator of the EMT signaling network [13]. We observed that changes in the expression levels of *circCORO1C* or *PBX3* affected the expression of EMT markers, indicating that the *circCORO1C*–*let-7c-5p*–*PBX3* axis promoted the migration and invasion of LSCC cells by regulating EMT.

Finally, we demonstrated that knockdown of *circCORO1C* inhibited the growth of LSCC cell xenograft tumors through preclinical models and verified the regulatory relationship of the *circCORO1C*–*let-7c-5p*–*PBX3* axis in vivo. In future, exploring the upstream regulator of *circCORO1C* and developing non-invasive *circCORO1C* detection methods in LSCC and other HNSCC types will be of great significance in promoting clinical translation.

## Conclusions

In summary, our data revealed that *circCORO1C* competitively binds *let-7c-5p* to eliminate its inhibitory effect on *PBX3*, thereby promoting LSCC cell proliferation, migration, and invasion (Fig. 8f). High expression of *circCORO1C* is an important marker of poor prognosis for LSCC. These findings provide new insights into the occurrence and progression of LSCC and indicate the potential of *circCORO1C* as a biomarker and therapeutic target for LSCC.

## Supplementary information

**Additional file 1: Table S1.** Clinical features of 57 LSCC samples for RNA sequencing. **Table S2.** Clinical features of 107 LSCC samples for qPCR validation. **Table S3.** Differentially expressed circRNAs in LSCC tissues. **Table S4.** Differentially expressed miRNAs in LSCC tissues. **Table S5.** Differentially expressed mRNAs in LSCC tissues. **Table S6.** Primer sequences for RT-PCR and qPCR analysis. **Table S7.** Prediction of *circCORO1C* and miRNA interaction by seedVicious. **Table S8.** *let-7c-5p* target gene prediction by ENCORI. **Table S9.** Intersection of predicted *let-7c-5p* targets and upregulated mRNAs in LSCC tissues

**Additional file 2: Figure S1.** RNA sequencing and high-content screening reveals that *circCORO1C* affects the proliferation of LSCC cells**. a** Flowchart showing the steps for identifying functional circRNAs in LSCC. **b** Validation of circRNA expression in LSCC tissues by RT-PCR and Sanger sequencing. **c** High-content screening of circRNAs that affect the proliferation of LSCC cells. GFP-labeled FD-LSC-1 cells were transfected with siRNAs targeting the indicated circRNA. After 24 h transfection, cells were seeded into 96-well plates, and the cell number was counted at the indicated time points. Representative images (left) and fold change in cell count (right) are shown. Data are presented as the means ± SD of three independent experiments. *P < 0.05. **Figure S2.** FD-LSC-1 cells were transfected with *let-7c-5p* mimics or NC mimics for 48 h, then RIP assay was performed using AGO2 antibody and *circCORO1C* levels were measured by qPCR. **P < 0.001.

## Data Availability

RNA sequencing raw data and normalized results were deposited at GEO database (GSE127165, GSE133632). All data that support the findings of this study are available from the corresponding authors upon reasonable request.

## Abbreviations

LSCC: Laryngeal squamous cell carcinoma
circRNA: Circular RNA
ceRNA: Competing endogenous RNA
EMT: Epithelial–mesenchymal transition
ANM: Adjacent normal mucosa
qPCR: Quantitative real-time PCR
FISH: Fluorescence in situ hybridization
EdU: 5-Ethynyl-2′-deoxyuridine
RIP: RNA immunoprecipitation
3′ UTR: 3′-untranslated region
HNSCC: Head and neck squamous cell carcinoma
