# Correction: Circular RNA *circCORO1C* promotes laryngeal squamous cell carcinoma progression by modulating the let-7c-5p/PBX3 axis

**DOI:** 10.1186/s12943-023-01756-4

**Published:** 2023-03-15

**Authors:** Yongyan Wu, Yuliang Zhang, Xiwang Zheng, Fengsheng Dai, Yan Lu, Li Dai, Min Niu, Huina Guo, Wenqi Li, Xuting Xue, Yunfeng Bo, Yujia Guo, Jiangbo Qin, Yixiao Qin, Hongliang Liu, Yu Zhang, Tao Yang, Li Li, Linshi Zhang, Rui Hou, Shuxin Wen, Changming An, Huizheng Li, Wei Xu, Wei Gao

**Affiliations:** 1 Shanxi Key Laboratory of Otorhinolaryngology Head and Neck Cancer, Shanxi Medical University, Taiyuan, 030001 Shanxi People’s Republic of China; 2 Shanxi Province Clinical Medical Research Center for Precision Medicine of Head and Neck Cancer, The First Hospital of Shanxi Medical University, Taiyuan, 030001 Shanxi People’s Republic of China; 3 Department of Otolaryngology Head & Neck Surgery, The First Hospital of Shanxi Medical University, Taiyuan, 030001 Shanxi People’s Republic of China; 4 Key Laboratory of Cellular Physiology, Ministry of Education, Shanxi Medical University, Taiyuan, 030001 Shanxi People’s Republic of China; 5 Department of Biochemistry & Molecular Biology, Shanxi Medical University, Taiyuan, 030001 Shanxi People’s Republic of China; 6 Department of Otolaryngology Head & Neck Surgery, The First Hospital, Jinzhou Medical University, Jinzhou, 121001 Liaoning People’s Republic of China; 7 Department of Pathology, Shanxi Cancer Hospital, Shanxi Medical University, Taiyuan, 030013 Shanxi People’s Republic of China; 8 Department of Otolaryngology Head & Neck Surgery, Heping Hospital Affiliated to Changzhi Medical College, Changzhi, 046000 Shanxi People’s Republic of China; 9 Department of Cell Biology and Genetics, Basic Medical School of Shanxi Medical University, Taiyuan, 030001 Shanxi People’s Republic of China; 10 Department of Physiology, Shanxi Medical University, Taiyuan, 030001 Shanxi People’s Republic of China; 11 Department of Hepatobiliary and Pancreatic Surgery, The Second Affiliated Hospital, Zhejiang University School of Medicine, Hangzhou, 310009 Zhejiang People’s Republic of China; 12 Harry Perkins Institute of Medical Research, QEII Medical Centre and Centre for Medical Research, the University of Western Australia, PO Box 7214, 6 Verdun Street, Nedlands, Perth, Western Australia 6009 Australia; 13 General Hospital, Shenzhen University, Shenzhen, 518055 Guangdong People’s Republic of China; 14 Department of Head and Neck Surgery, Cancer Hospital, National Cancer Center, Chinese Academy of Medical Sciences & Peking Union Medical College, Beijing, 100021 People’s Republic of China; 15 Department of Otolaryngology Head & Neck Surgery, Dalian Municipal Friendship Hospital, Dalian Medical University, Dalian, 116100 Liaoning People’s Republic of China; 16 Shandong Provincial ENT Hospital Affiliated to Shandong University, Jinan, 250022 Shandong People’s Republic of China; 17 Shandong Provincial Institute of Otolaryngology, Jinan, 250022 Shandong People’s Republic of China; 18 Key Laboratory of Otolaryngology, Ministry of Health, Shandong University, Jinan, 250022 Shandong People’s Republic of China

**Correction: Mol Cancer 19, 99 (2020)**

**https://doi.org/10.1186/s12943-020-01215-4**

In our research published [1] in *Molecular Cancer* entitled “Circular RNA *circCORO1C* promotes laryngeal squamous cell carcinoma progression by modulating the let-7c-5p/PBX3 axis” (Molecular Cancer 19, Article number: 99 (2020)), we identified minor errors in the images presented in Figs. 5E and 6L recently. Specifically, overlap was found in the representative migration images between the “TU-177 let-7c-5p inhibitor” group of Fig. 5E (row 3, column 3) and the “TU-177 NC” group of Fig. 6L (row 1, column 1). We have double-checked the original data and found that the inadvertent errors occurred during picture compilation. Unfortunately, this error was not found during the submission and proof stages.

**Fig. 5:**
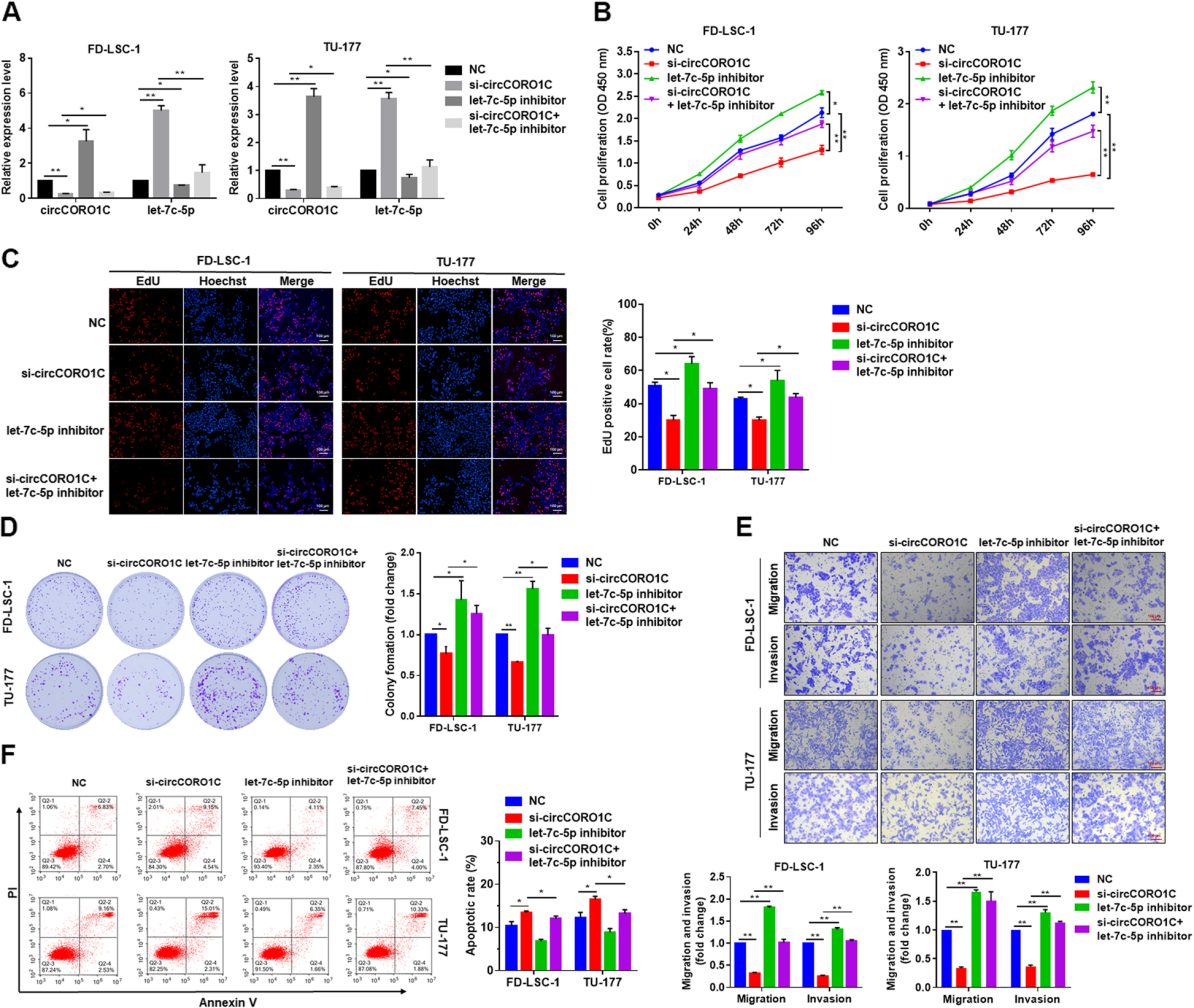
*let-7c-5p* reversed the tumor-promoting effect of *circCORO1C* in LSCC cells. **a** FD-LSC-1 and TU-177 cells were transfected with si-*circCORO1C* or co-transfected with si-*circCORO1C* and *let-7c-5p* inhibitor. *CircCORO1C* and *let-7c-5p* expression was detected by qPCR. **b** FD-LSC-1 and TU-177 cells were transfected with si-*circCORO1C* or co-transfected with si-*circCORO1C* and *let-7c-5p* inhibitor. Cell proliferation was determined by CCK8 assay. **c** Effects of si-*circCORO1C* and *let-7c-5p* inhibitor on the proliferation of FD-LSC-1 and TU-177 cells were evaluated by EdU staining. **d** Colony formation assays were performed to evaluate the proliferative ability of FD-LSC-1 and TU-177 cells transfected with si-*circCORO1C* or co-transfected with si-*circCORO1C* and *let-7c-5p* inhibitor. **e** Effects of si-*circCORO1C* and *let-7c-5p* inhibitor on the migration and invasion of FD-LSC-1 and TU-177 cells were evaluated by Transwell migration and invasion assays. **f** FD-LSC-1 and TU-177 cells were transfected with si-*circCORO1C* or co-transfected with si-*circCORO1C* and *let-7c-5p* inhibitor*.* Cells were stained with Annexin V-FITC and PI, and the percentage of apoptotic cells was detected by flow cytometry. Data are presented as the means ± SD of three independent experiments. *P < 0.05; **P < 0.001

**Fig. 6:**
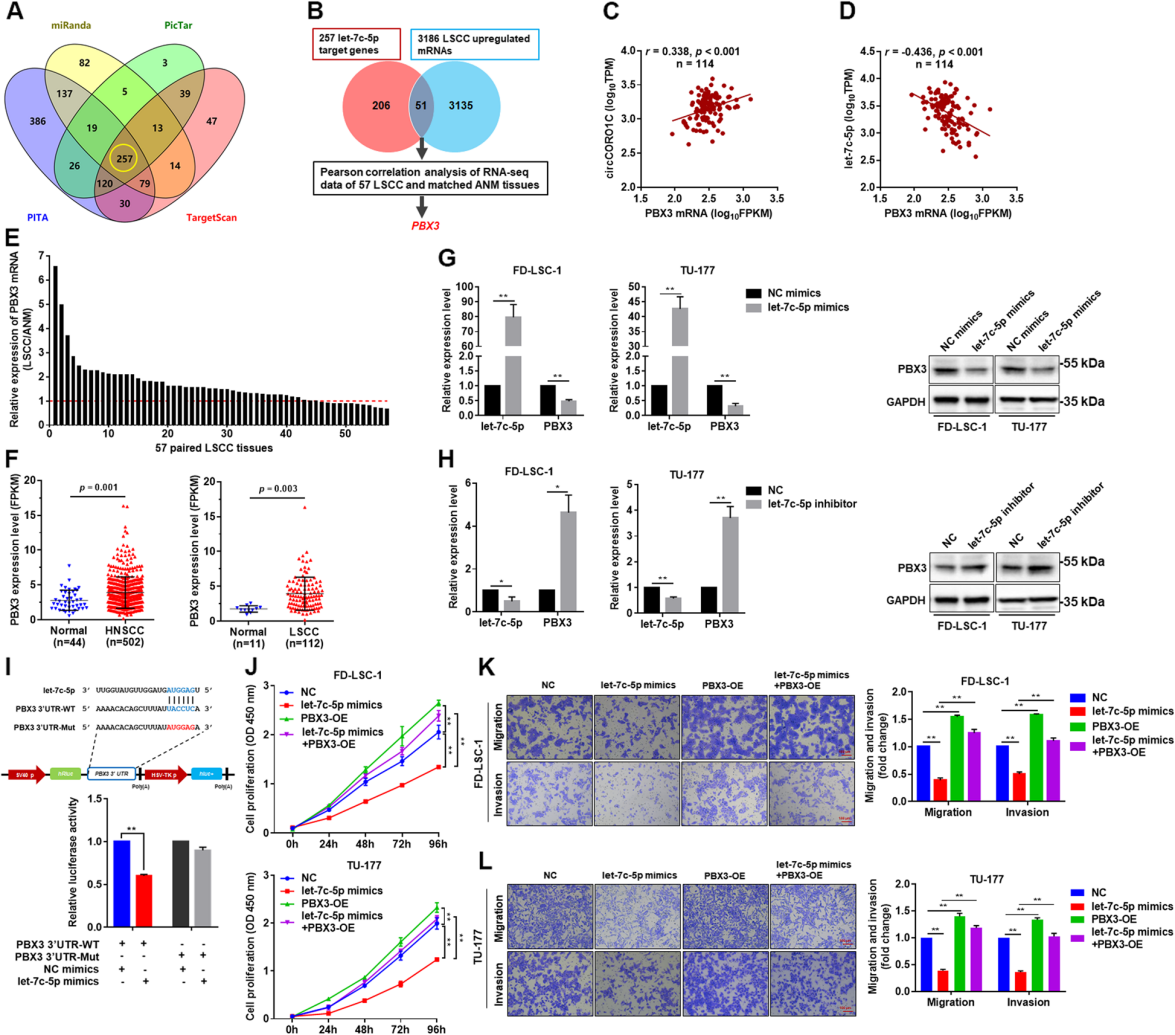
*PBX3* is a direct target gene of *let-7c-5p*, which acted as an oncogene in LSCC cells. **a** Venn analysis of the target genes of *let-7c-5p* predicted by miRanda, PicTar, PITA, and TargetScan. **b** Integrated analysis of bioinformatics-predicted target genes and RNA sequencing data of 57 pairs of LSCC tissues was performed to screen for *let-7c-5p* target genes. **c** & **d** Correlation analysis between *circCORO1C* (**c**) or *let-7c-5p* (**d**) and *PBX3* expression using RNA sequencing data of 57 pairs of LSCC tissues and matched ANM tissues. **e ***PBX3* expression in RNA sequencing data of 57 pairs of LSCC tissues and matched ANM tissues. The expression levels of *PBX3* in each LSCC tissue were normalized to corresponding matched ANM tissue. **f** Analysis of *PBX3* expression in HNSCC and LSCC tissues using transcriptome sequencing data from TCGA database. **g** & **h** FD-LSC-1 and TU-177 cells were transfected with *let-7c-5p* mimics (**g**), *let-7c-5p* inhibitor (**h**) or NC, and PBX3 expression was detected by qPCR and western blotting. **i** HEK293T cells were co-transfected with *let-7c-5p* mimics and wild-type or mutant *PBX3* 3′ UTR reporter plasmids, and luciferase reporter assays were performed to evaluate the effect of *let-7c-5p* on luciferase activity. **j** FD-LSC-1 and TU-177 cells were transfected with *let-7c-5p* mimics or co-transfected with *let-7c-5p* mimics and *PBX3* overexpression plasmids, and CCK8 assay was performed to detect cell proliferation. **k** & **l** FD-LSC-1 (**k**) and TU-177 (**l**) cells were transfected with *let-7c-5p* mimics or co-transfected with *let-7c-5p* mimics and *PBX3* overexpression plasmids. Changes in cell migration and invasion capacity were evaluated by Transwell assays. Data are presented as the means ± SD of three independent experiments. *P < 0.05; **P < 0.001

The corrected Figs. 5E, 6L are attached, and the correction does not change the results and scientific conclusions of this article. We sincerely apologize to the editor, reviewers and readers for the errors and any confusion it may have caused. We want to make a correction to this error as soon as possible.
