# Correction: Circular RNA *circCORO1C* promotes laryngeal squamous cell carcinoma progression by modulating the let-7c-5p/ PBX3 axis

**DOI:** 10.1186/s12943-023-01819-6

**Published:** 2023-07-12

**Authors:** Yongyan Wu, Yuliang Zhang, Xiwang Zheng, Fengsheng Dai, Yan Lu, Li Dai, Min Niu, Huina Guo, Wenqi Li, Xuting Xue, Yunfeng Bo, Yujia Guo, Jiangbo Qin, Yixiao Qin, Hongliang Liu, Yu Zhang, Tao Yang, Li Li, Linshi Zhang, Rui Hou, Shuxin Wen, Changming An, Huizheng Li, Wei Xu, Wei Gao

**Affiliations:** 1 Shanxi Key Laboratory of Otorhinolaryngology Head and Neck Cancer, Shanxi Medical University, Taiyuan, 030001 Shanxi People’s Republic of China; 2 Shanxi Province Clinical Medical Research Center for Precision Medicine of Head and Neck Cancer, The First Hospital of Shanxi Medical University, Taiyuan, 030001 Shanxi People’s Republic of China; 3 Department of Otolaryngology Head & Neck Surgery, The First Hospital of Shanxi Medical University, Taiyuan, 030001 Shanxi People’s Republic of China; 4 Key Laboratory of Cellular Physiology, Ministry of Education, Shanxi Medical University, Taiyuan, 030001 Shanxi People’s Republic of China; 5 Department of Biochemistry & Molecular Biology, Shanxi Medical University, Taiyuan, 030001 Shanxi People’s Republic of China; 6 Department of Otolaryngology Head & Neck Surgery, The First Hospital, Jinzhou Medical University, Jinzhou, 121001 Liaoning People’s Republic of China; 7 Department of Pathology, Shanxi Cancer Hospital, Shanxi Medical University, Taiyuan, 030013 Shanxi People’s Republic of China; 8 Department of Otolaryngology Head & Neck Surgery, Heping Hospital Affiliated to Changzhi Medical College, Changzhi, 046000 Shanxi People’s Republic of China; 9 Department of Cell Biology and Genetics, Basic Medical School of Shanxi Medical University, Taiyuan, 030001 Shanxi People’s Republic of China; 10 Department of Physiology, Shanxi Medical University, Taiyuan, 030001 Shanxi People’s Republic of China; 11 Department of Hepatobiliary and Pancreatic Surgery, The Second Affiliated Hospital, Zhejiang University School of Medicine, Hangzhou, 310009 Zhejiang People’s Republic of China; 12 Harry Perkins Institute of Medical Research, QEII Medical Centre and Centre for Medical Research, University of Western Australia, 6 Verdun Street, Nedlands, PO Box 7214, Perth, WA 6009 Australia; 13 General Hospital, Shenzhen University, Shenzhen, 518055 Guangdong People’s Republic of China; 14 Department of Head and Neck Surgery, Cancer Hospital, National Cancer Center, Chinese Academy of Medical Sciences & Peking Union Medical College, Beijing, 100021 People’s Republic of China; 15 Department of Otolaryngology Head & Neck Surgery, Dalian Municipal Friendship Hospital, Dalian Medical University, Dalian, 116100 Liaoning People’s Republic of China; 16 Shandong Provincial, ENT Hospital Affiliated to Shandong University, Jinan, 250022 Shandong People’s Republic of China; 17 Shandong Provincial Institute of Otolaryngology, Jinan, 250022 Shandong People’s Republic of China; 18 Key Laboratory of Otolaryngology, Ministry of Health, Shandong University, Jinan, 250022 Shandong People’s Republic of China

**Correction: Mol Cancer 19, 99 (2020)**

**https://doi.org/10.1186/s12943-020-01215-4**

Following publication of the original article [1], the authors noted that the sequence of let-7c-5p in Fig. 3C and 6I is correct, while the let-7c-5p primer sequence error appears in Additional file 1: Table S6. The corrected Table S6 has been included in this correction.

## Supplementary Information

**Additional file 1:** **Table S6.** Primer sequences for RT-PCR and qPCR analysis.
