# Supplementary material for: Circular RNA circCORO1C promotes laryngeal squamous cell carcinoma progression by modulating the let-7c-5p/PBX3 axis
Source: Mol Cancer. 2020 Jun 2;19:99. doi: 10.1186/s12943-020-01215-4 (PMC7265647; doi:10.1186/s12943-020-01215-4)
Supplement: Supplementary file 1 — Additional file 1: Table S1. Clinical features of 57 LSCC samples for RNA sequencing. Table S2. Clinical features of 107 LSCC samples for qPCR validation. Table S3. Differentially expressed circRNAs in LSCC tissues. Table S4. Differentially expressed miRNAs in LSCC tissues. Table S5. Differentially expressed mRNAs in LSCC tissues. Table S6. Primer sequences for RT-PCR and qPCR analysis. Table S7. Prediction of circCORO1C and miRNA interaction by seedVicious. Table S8. let-7c-5p target gene prediction by ENCORI. Table S9. Intersection of predicted let-7c-5p targets and upregulated mRNAs in LSCC tissues [file 12943_2020_1215_MOESM1_ESM.zip › Table S6. Primer sequences for RT-PCR and qPCR analysis.docx]

| **Primer name** | **Forward (5’ - 3’)** | **Reverse (5’ - 3’)** |
| --- | --- | --- |
| circCORO1C divergent | AAGGGTGACAGCAGTATTC | GTCATAGAAAGGCAGCAAC |
| circCORO1C convergent | GGTGACAGCAGTATTCGC | CTGGCAATCTCACATTTG |
| CORO1C | CTCAATTGCCTGCCAAGGGT | TTCACCGCTTGCCCAAATAC |
| GAPDH divergent | CATGGCCCACATGGCCTCC | GTTCTCAGCCTTGACGGT |
| GAPDH convergent | GCTCTCTGCTCCTCCTGTTC | ACGACCAAATCCGTTGACTC |
| let-7c-5p | TGAGGTAGTAGGTTGTGTGGTT | Universal primer provided by miRNA First-Strand cDNA Synthesis Kit |
| PBX3 | GTTCGCGTCGCGTCTGCAGTG | TGTTTCCGTGGGAAGTCAACA |
| 18s rRNA | CCTGGATACCGCAGCTAGGA | GCGGCGCAATACGAATGCCCC |
| U6 RNA | ATTTGCGTGTCATCCTTGC | TCGCTTCGGCAGCACATAT |

**Table S6. Primer sequences for RT-PCR and qPCR analysis.**
