# Supplementary figures and images for: Circular RNA circCORO1C promotes laryngeal squamous cell carcinoma progression by modulating the let-7c-5p/PBX3 axis
Source: Mol Cancer. 2020 Jun 2;19:99. doi: 10.1186/s12943-020-01215-4 (PMC7265647; doi:10.1186/s12943-020-01215-4)

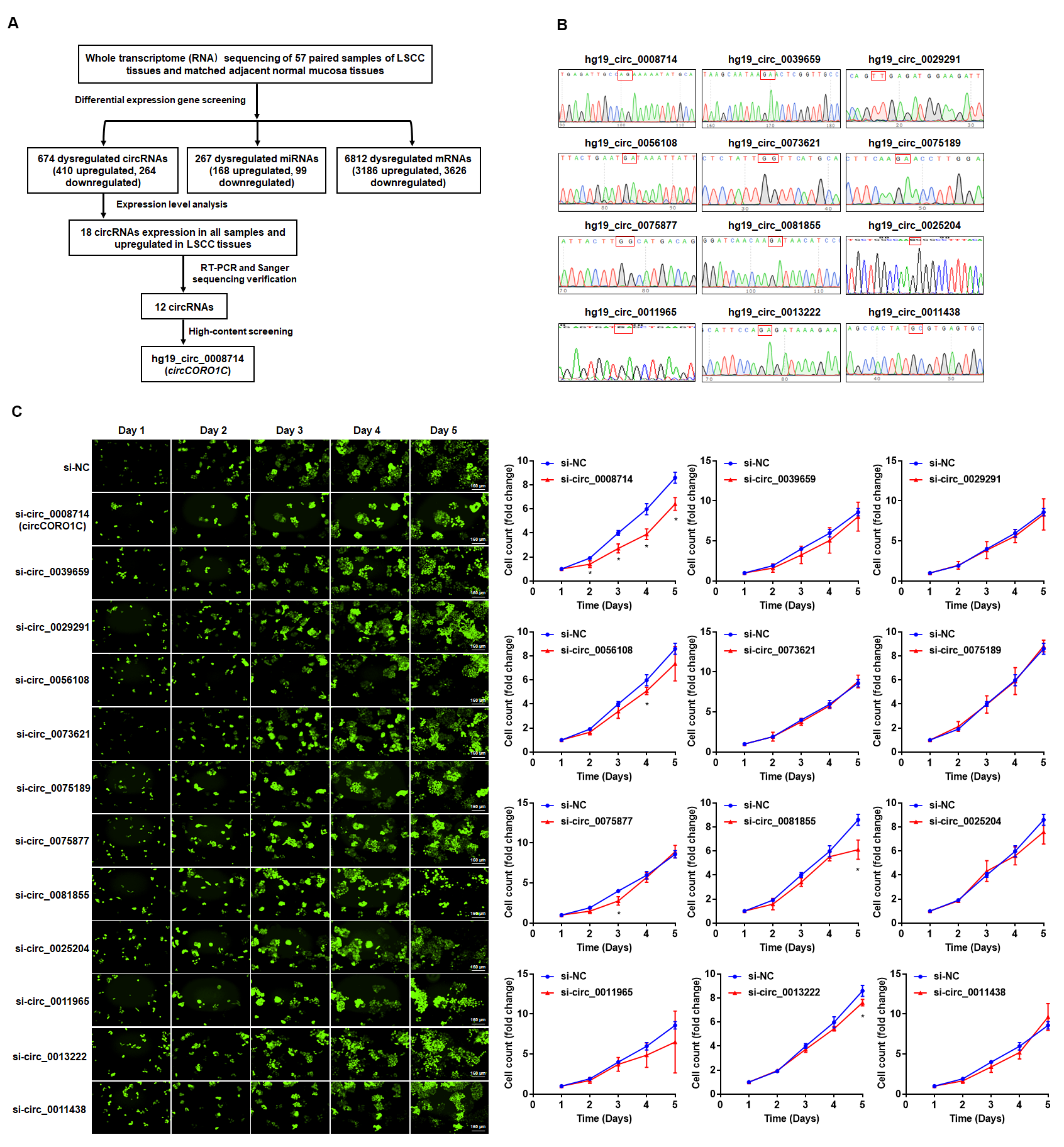

Supplement: Supplementary file 2 — Additional file 2: Figure S1. RNA sequencing and high-content screening reveals that circCORO1C affects the proliferation of LSCC cells. a Flowchart showing the steps for identifying functional circRNAs in LSCC. b Validation of circRNA expression in LSCC tissues by RT-PCR and Sanger sequencing. c High-content screening of circRNAs that affect the proliferation of LSCC cells. GFP-labeled FD-LSC-1 cells were transfected with siRNAs targeting the indicated circRNA. After 24 h transfection, cells were seeded into 96-well plates, and the cell number was counted at the indicated time points. Representative images (left) and fold change in cell count (right) are shown. Data are presented as the means ± SD of three independent experiments. *P < 0.05. Figure S2. FD-LSC-1 cells were transfected with let-7c-5p mimics or NC mimics for 48 h, then RIP assay was performed using AGO2 antibody and circCORO1C levels were measured by qPCR. **P < 0.001. [file 12943_2020_1215_MOESM2_ESM.zip › Figure S1.tif]

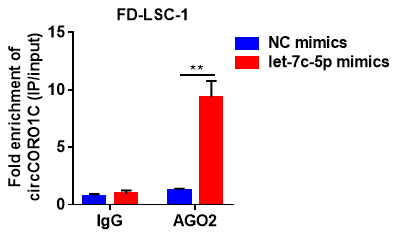

Supplement: Supplementary file 2 — Additional file 2: Figure S1. RNA sequencing and high-content screening reveals that circCORO1C affects the proliferation of LSCC cells. a Flowchart showing the steps for identifying functional circRNAs in LSCC. b Validation of circRNA expression in LSCC tissues by RT-PCR and Sanger sequencing. c High-content screening of circRNAs that affect the proliferation of LSCC cells. GFP-labeled FD-LSC-1 cells were transfected with siRNAs targeting the indicated circRNA. After 24 h transfection, cells were seeded into 96-well plates, and the cell number was counted at the indicated time points. Representative images (left) and fold change in cell count (right) are shown. Data are presented as the means ± SD of three independent experiments. *P < 0.05. Figure S2. FD-LSC-1 cells were transfected with let-7c-5p mimics or NC mimics for 48 h, then RIP assay was performed using AGO2 antibody and circCORO1C levels were measured by qPCR. **P < 0.001. [file 12943_2020_1215_MOESM2_ESM.zip › Figure S2.tif]
