# Supplementary material for: Correction: Circular RNA circCORO1C promotes laryngeal squamous cell carcinoma progression by modulating the let-7c-5p/ PBX3 axis
Source: Mol Cancer. 2023 Jul 12;22:109. doi: 10.1186/s12943-023-01819-6 (PMC10337073; doi:10.1186/s12943-023-01819-6)
Supplement: Supplementary file 1 — Additional file 1: Table S6. Primer sequences for RT-PCR and qPCR analysis. [file 12943_2023_1819_MOESM1_ESM.docx]

Table S6. Primer sequences for RT-PCR and qPCR analysis.

| **Primer name** | **Forward (5’ - 3’)** | **Reverse (5’ - 3’)** |
| --- | --- | --- |
| circCORO1C divergent | AAGGGTGACAGCAGTATTC | GTCATAGAAAGGCAGCAAC |
| circCORO1C convergent | GGTGACAGCAGTATTCGC | CTGGCAATCTCACATTTG |
| CORO1C | CTCAATTGCCTGCCAAGGGT | TTCACCGCTTGCCCAAATAC |
| GAPDH divergent | CATGGCCCACATGGCCTCC | GTTCTCAGCCTTGACGGT |
| GAPDH convergent | GCTCTCTGCTCCTCCTGTTC | ACGACCAAATCCGTTGACTC |
| let-7c-5p | TGAGGTAGTAGGTTGTATGGTT | Universal primer provided by miRNA  First-Strand cDNA Synthesis Kit |
| PBX3 | GTTCGCGTCGCGTCTGCAGTG | TGTTTCCGTGGGAAGTCAACA |
| 18s rRNA | CCTGGATACCGCAGCTAGGA | GCGGCGCAATACGAATGCCCC |
| U6 RNA | ATTTGCGTGTCATCCTTGC | TCGCTTCGGCAGCACATAT |
